# Deep biochemical phenotyping reveals prognostic value of rare genetic variants in adult kidney stone disease

**DOI:** 10.1172/JCI196277

**Published:** 2026-03-02

**Authors:** Johannes Münch, Jana Petrovska, Joana Figueiro-Silva, Isabel Rubio-Aliaga, Elena M. Cabello, Ivan Ivanovski, Michael Papik, Beatrice Oneda, Daniel G. Fuster, Harald Seeger, Thomas Ernandez, Florian Buchkremer, Gregoire Wuerzner, Nasser A. Dhayat, Alexander Ritter, Stephan Segerer, Beat Roth, Anita Rauch, Pietro Manuel Ferraro, Olivier Bonny, Carsten A. Wagner, Ruxandra Bachmann-Gagescu

**Affiliations:** 1 Institute of Medical Genetics, University of Zurich, Schlieren-Zurich, Switzerland.; 2 Institute of Physiology, and; 3 Department of Molecular Life Sciences, University of Zurich, Zurich, Switzerland.; 4 National Center of Competence in Research (NCCR) Kidney.CH, Bern, Switzerland.; 5 Department of Nephrology and Hypertension, Inselspital, Bern University Hospital, University of Bern, Bern, Switzerland.; 6 Department of Nephrology, University Hospital Zurich, Zurich, Switzerland.; 7 Service of Nephrology, Geneva University Hospitals, Geneva, Switzerland.; 8 Division of Nephrology, Cantonal Hospital Aarau, Aarau, Switzerland.; 9 Service of Nephrology and Hypertension, Lausanne University Hospital and University of Lausanne, Lausanne, Switzerland.; 10 Department of Urology, University Hospital of Bern, Inselspital, University of Bern, Bern, Switzerland.; 11 Section of Nephrology, Department of Medicine, Università degli Studi di Verona, Verona, Italy.; 12 Department of Biomedical Sciences, University of Lausanne, Lausanne, Switzerland.; 13 Zurich Kidney Center, University of Zurich, Zurich, Switzerland.

**Keywords:** Genetics, Nephrology, Monogenic diseases

## Abstract

**BACKGROUND:**

Kidney stone disease (KSD) affects approximately 10% of the population. While genetic factors are known to play a role in KSD, determining the clinical relevance of rare variants in KSD genes identified in adults remains challenging.

**METHODS:**

The Swiss Kidney Stone Cohort is a multicenter longitudinal, observational study consisting of kidney stone formers (KSFs) (*n* = 701) and non-kidney stone formers (NKSFs) (*n* = 200). Blood and urine samples were collected at enrollment and over 3 years for deep biochemical phenotyping. Results were correlated with rare genetic variants in established KSD genes identified through whole-exome sequencing and classified according to American College of Medical Genetics and Genomics and the Association of Molecular Pathology (ACMG/AMP) criteria.

**RESULTS:**

Collectively, we found rare (likely) pathogenic (LP/P) variants representing strong KSD risk factors in 6.8% of KSFs, predominantly in genes involved in renal phosphate handling and cystinuria. Detailed biochemical analyses confirmed that KSFs carrying heterozygous LP/P *SLC34A3* variants exhibited significant hyperphosphaturia. In contrast, monoallelic LP/P variants in *SLC34A1*, *SLC9A3R1*, or *CYP24A1*, which were also frequent in NKSFs, did not result in the expected biochemical alterations, calling into question their causative role as strong KSD risk factors. In cystinuria, monoallelic *SLC7A9* variants represented intermediate risk factors, since they caused biochemical alterations but required additional factors for KSD occurrence, based on frequent LP/P variants in NKSFs. The presence of strong risk factors was associated with higher kidney stone (KS) recurrence over the 3-year observation period, supporting a predictive value for genetic testing.

**CONCLUSIONS:**

Correlation of genetic findings with thorough biochemical phenotyping and comparison with NKSFs redefines the clinical relevance of variants in KSD genes and has prognostic value.

## Abstract 2

Integrating blood and urine biochemistry with genetics improves interpretation of genetic findings in adults with kidney stone disease and has prognostic value, enabling personalized risk assessment.

## Introduction

Kidney stone disease (KSD) has a lifetime prevalence of approximately 10% and ranks among the most common diseases worldwide (1). Patients experience severe short-term symptoms and may have an increased long-term risk for chronic kidney disease (CKD) and progression to end-stage kidney failure (2, 3). KSD results from urine supersaturation due to an imbalance between crystallization promoters (e.g. calcium, oxalate, phosphate, cystine, or urate) and inhibitors (e.g., magnesium and citrate). The pathogenesis of KSD is multifactorial, including dietary factors, metabolic alterations, and genetic predisposition. The role of genetic factors ranges from “monogenic KSD,” in which genetic variants in a single gene are sufficient to cause disease, to complex polygenic disease (e.g., presence of SNPs), in which other risk factors (e.g., lifestyle) play a more important role and are required in addition to genetic predisposing factors (4–6).

Monogenic disorders are defined by the presence of mono- or biallelic likely pathogenic (LP) or pathogenic (P) variants in a single gene that cause the respective disease in the majority of (or even in all) cases following a Mendelian (dominant, recessive, or X-linked) inheritance mode. In monogenic disorders, identification of LP/P variants allows for precise calculation of the risk for offspring and family members to inherit the relevant genetic change and hence to develop the disease. The presence of such genetic variants typically has sufficient predictive value regarding disease occurrence to have implications for family planning and to warrant genetic counseling and consideration of genetic testing of unaffected family members. Among the genes most commonly associated with monogenic KSD are those involved in renal phosphate handling (*SLC34A1*, *SLC34A3*, *SLC9A3R1*) or cystine excretion (*SLC7A9*, *SLC3A1*) (7, 8). Dysfunction of the encoded transporters leads to increased urinary concentration of crystallizing factors constituting such a strong genetic risk factor, that kidney stones (KSs) are very likely to occur. Beyond implications for genetic counseling, identification of pathogenic variants associated with a diagnosis of monogenic KSD can lead to targeted therapeutic interventions (9, 10).

At the other end of the spectrum, most cases of KSD are not directly caused by defects in a single gene, but genetic factors likely contribute to disease risk, based on clustering of KSD within families, without following Mendelian inheritance (11–13). However, this dichotomy between monogenic KSD and complex KSD is probably somewhat artificial, as it appears more likely that the relative contribution of environmental and genetic factors represents a continuum rather than a clear-cut distinction (6, 11). Moreover, several GWASs have identified common polymorphisms that are associated with KSD, in which individual variants have little effect alone but together confer an increased risk for KSD (14–16). It may therefore be more relevant to classify genetic variants as strong, intermediate-, or low-risk factors.

Previous studies have reported very different rates when studying monogenic KSD, which is in part explained by differences in the study populations. In pediatric and preselected cohorts, such monogenic KSD has been shown to account for up to 7%–30% (17–21), whereas in unselected cohorts, this rate is lower (2.7%–8%) (22, 23). Such reported “solve rates” for monogenic KSD are, however, strongly influenced not only by the characteristics of the cohort analyzed, but also by the criteria used to designate variants as disease-causing. Indeed, prediction of the pathogenicity of genetic variants remains a major challenge in medical genetics, despite established classification frameworks such as the American College of Medical Genetics and Genomics and the Association of Molecular Pathology (ACMG/AMP) guidelines (24). This classification was developed for rare monogenic diseases and may not be appropriate for more common diseases such as KSD. Moreover, the majority of novel missense variants identified are classified as variants of uncertain significance (VUS), which is of limited utility in making decisions about patient management and counseling. Even when variants have been previously described and classified as LP/P or likely benign or benign (LB/B), this interpretation is not always reliable and/or may be incongruent with the observed carrier frequencies, in some cases exceeding 1% for LP/P variants (25), suggesting either misclassification or highly incomplete penetrance. Highly incomplete penetrance is in fact another way of describing genetic variants that require additional factors (genetic or environmental) to result in disease manifestation, illustrating the fact that the genetics of KSD challenge the classical monogenic-complex disease classification. In addition, the fact that some genes such as *SLC34A1* have been suggested to cause KSD in both recessive and dominant modes (26), further complicates the interpretation of the clinical significance of single heterozygous variants in such genes. For some genes, such as *SLC34A3* or *CYP24A1*, biallelic pathogenic variants result in recessive diseases, hypophosphatemic rickets, and infantile hypercalcemia, respectively, while monoallelic pathogenic variants have been suggested to cause milder forms of disease with an increased risk for KSs (27, 28). Taken together, the current challenges in variant classification (pathogenic or not) and the question of the applicable inheritance mode for a given gene/variant (recessive and/or dominant) substantially complicate interpretation of the clinical relevance of variants in established KSD genes. Moreover, even when reaching the conclusion that a patient has monogenic KSD, the prognostic value of this information has not yet been proven.

To incorporate results from genetic diagnostics into patient care, clinicians require reliable genotype-phenotype descriptions. Large genetic databases often lack detailed phenotypic data and long-term follow-up information. To address these gaps, we used a unique large and biochemically deeply phenotyped cohort of adult patients with KSs with 3-year follow-up to integrate detailed biochemical analysis into genetic data interpretation. We further compared the genetic variants identified in kidney stone formers (KSFs) with those of proven non–kidney stone formers (NKSFs). This approach allowed us to reinterpret the significance of selected monoallelic genetic variants, confirming some as strong risk factors while others were ruled out as directly causative for KSD, and to evaluate the prognostic relevance of such “monogenic” KSD with strong genetic risk factors.

## Results

### Cohort characteristics and overall genetic findings

**Figure 1:**
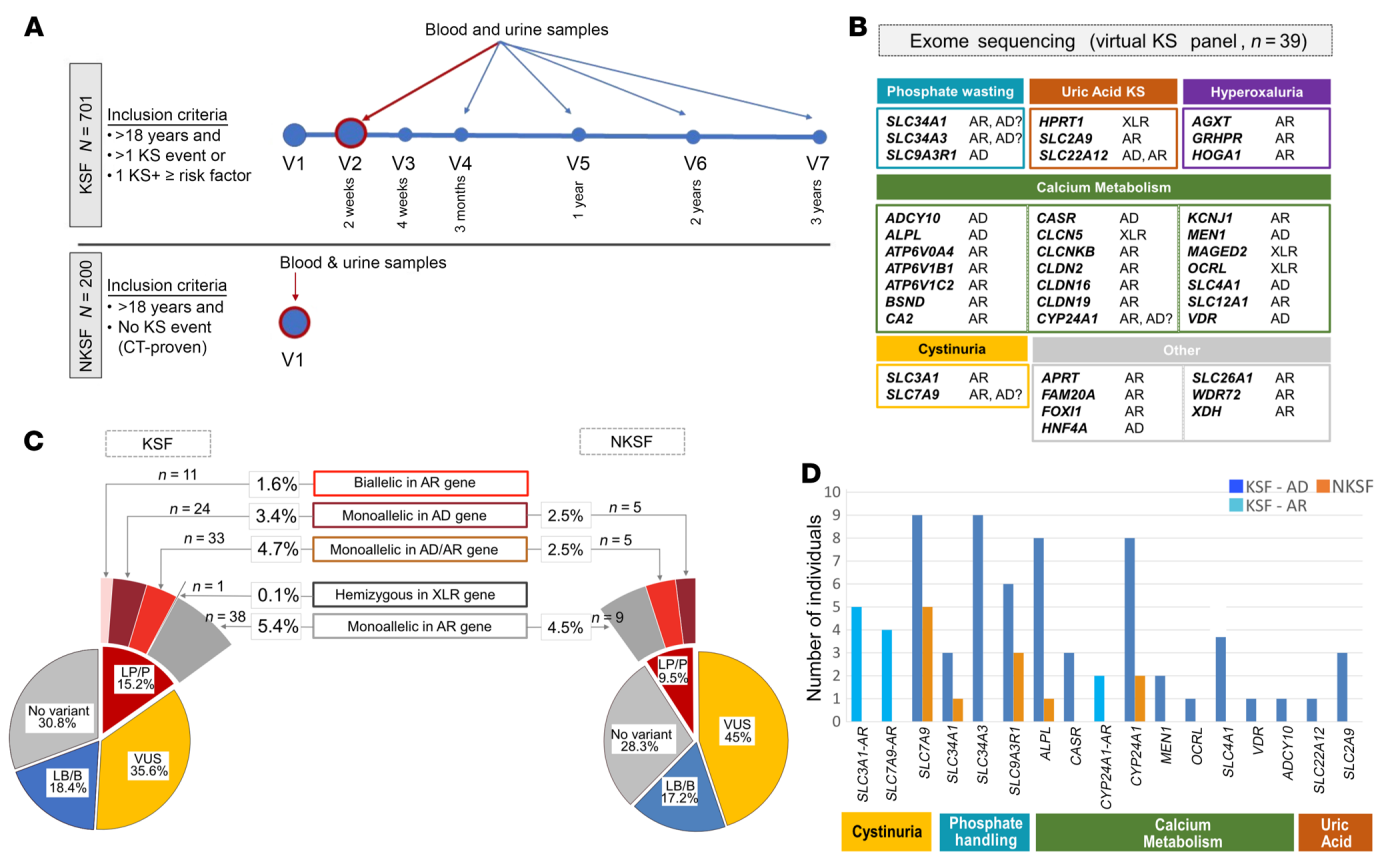
Variants in monogenic KS genes identified in the SKSC. (**A**) Overview of the study and inclusion criteria (*see also Supplemental Figure 1 for detailed risk factors). v indicates the visit number, with 7 visits (V1–7) over 3 years for KSFs, and 1 visit (V1) for NKSFs. Visits with measurement of biochemical parameters (blood and urine samples) are indicated above with arrows (values from the baseline visit [V2, red arrow] were used in the following analyses in Figures 2 and 3). (**B**) WES was performed for KSFs and NKSFs, with analysis of a virtual panel for 39 established KS genes that are involved in the handling of different electrolytes. The described inheritance mode for each gene is indicated as autosomal recessive (AR), autosomal dominant (AD) or X-linked recessive (XLR), with the question mark indicating where the dominant inheritance mode has been debated. (**C**) Pie charts indicate the number of individuals harboring variants classified according to ACMG/AMP criteria for KSFs (left) and NKSFs (right). Each individual is accounted for only once for this plot. (**D**) The number of KSFs (blue) and NKSFs (orange) carrying a LP/P variant in 1 of the 39 KSD genes, with a matching mode of inheritance resulting in the diagnosis of monogenic KSD. Light blue bars indicate recessive disease ([presumed] biallelic variants in the indicated gene), and dark blue bars indicate dominant disease (monoallelic variants). No NKSF presented with biallelic LP/P variants. One KSF harbored a monoallelic LP/P variant in *SLC9A3R1* together with 2 LP/P variants in *SLC7A9*, while 1 NKSF carried a monoallelic LP/P variant in 2 genes associated with dominant and recessive disease (*SLC9A3R1* and *SLC7A9*). These individuals are indicated twice in the bar plot. All details for the LP/P variants are described in Supplemental Table 2 for KSFs and Supplemental Table 3 for NKSFs. AD, autosomal dominant; AR, autosomal recessive; B, benign; CT, computed tomography; KS, kidney stones; KSF, kidney stone formers; LB, likely benign; LP, likely pathogenic; NKSF, non-kidney stone formers; P, pathogenic, VUS, variant of uncertain significance, XLR, X-linked recessive.

This study analyzed data from 701 KSF and 200 NKSF participants from the Swiss Kidney Stone Cohort (SKSC), a multicentric, longitudinal observational study. Details on enrollment criteria, cohort population, and study design are described in Bonny et al. (29) and shown in Figure 1A and Supplemental Figure 1; supplemental material available online with this article; https://doi.org/10.1172/JCI196277DS1 In summary, KSFs aged 18 years or older with a recent stone event (at least 4 weeks but not more than 3 months before visit 1) were screened for inclusion. Referrals proceeded mostly (but not exclusively) through urology departments. Inclusion criteria included 2 or more KS events (at least 3 months apart) or 1 KS event (before age 25 years) plus 1 or more additional risk factors (see Supplemental Figure 1 for complete list of risk factors). These risk factors were not taken into account for enrollment of NKSFs, who were selected on the basis of an absence of KSs (history and CT-proven). Given the broad inclusion criteria and the lack of exclusion criteria for KSFs (beyond lack of consent and age <18 years), this cohort can be considered a relatively unselected cohort of adults with a higher risk for recurrent KSs representative of adult KSFs.

Comparison of demographics between KSF and NKSF cohorts showed a predominantly European ancestry and a male predominance in both cohorts but more pronounced in the KSFs, with slightly younger age for NKSFs (mean 46 ± 13 years in NKSFs versus 53 ± 14 years in KSFs) (Table 1). Consistent with previous knowledge, a positive family history of KSD was more frequently found in KSFs than in NSKFs, as was the prevalence of hypertension and diabetes (Table 1). Mean blood and 24-hour urine parameters were within normal reference ranges, however, KSF participants had slightly lower calcium (adjusted for albumin) and 25-OH vitamin D_3_ (but not 1,25-(OH)_2_ vitamin D_3_), as well as higher parathyroid hormone (PTH) and uric acid plasma values than did NKSFs. Excretion of the urinary crystallization inhibitors citrate and magnesium was lower in KSFs, whereas urinary calcium excretion was higher than in NKSFs (statistical significance not withstanding correction for multiple testing except for urinary calcium and citrate) (Table 1). Surprisingly, urine oxalate excretion was significantly lower in KSFs than in NKSFs. This may be explained by dietary adjustments made by study participants after the occurrence of earlier KS events. KS composition was predominantly calcium oxalate in approximately two-thirds of cases, with the remaining third having a variety of other compositions (uric acid, calcium phosphate, struvite, brushite or cystine) and many stones showing mixed compositions (Supplemental Figure 2). Whole-exome sequencing (WES) was performed on all KSF and NKSF participants (Supplemental Figure 3). Ancestry analysis was performed on the WES data for individuals with European ancestry, showing a highly mixed distribution of European ancestries (Supplemental Figure 4), consistent with sociodemographic knowledge of the Swiss population. Variants were called in 39 known KSD genes commonly tested as gene panels in individuals with suspected monogenic causes of KSD (6) (Figure 1B and Supplemental Table 1). Only coding nonsynonymous variants with a mean allele frequency (MAF) of less than 0.01 in the Genome Aggregation Database (gnomAD), canonical splice-site variants, and a few intronic variants previously described and well established as pathogenic were included in our analysis.

Taking the approach to identify individuals with monogenic KSD, we classified all identified rare variants in KSFs and NKSFs as LP/P, LB/B, or VUS using the ACMG/AMP criteria (24) and took into consideration the previously ascribed inheritance mode for each gene. We found that 15.2% (*n* = 107) of KSFs carried at least 1 LP/P variant in 1 or more of the analyzed genes. In comparison, 9% (*n* = 18) of NKSFs also carried at least 1 LP/P variant, resulting in a statistically significant higher rate of LP/P variants in KSFs than in NKSFs (*P* = 0.0238, χ^2^) (Figure 1C). Supplemental Table 2 summarizes the phenotypic description (age at first KS event, stone composition, EQUIL2-based urinary supersaturation scores for lithogenic substances) and variant classification of all KSFs with LP/P variants, and Supplemental Table 3 summarizes this information for NKSFs.

#### Recessive and X-linked KSD.

We found that 1.6% of KSFs (*n* = 11) carried homozygous/presumed biallelic LP/P variants in a gene associated with autosomal recessive (AR) KSD (*SLC3A1*, *CYP24A1*) or in a gene reported to cause KSD in autosomal dominant (AD) and recessive (AR) modes (*SLC7A9*) (Figure 1, C and D). In 5 of 11 cases, the variants were homozygous, and in 3 of 11 cases, next-generation sequencing (NGS) read data demonstrated that the variants were in *trans* (compound heterozygous), whereas for the remaining 3 of 11 KSF, the variants were too far apart to use NGS reads for segregation and family members were not available. Using the biochemical data available, we found that 2 of these 3 KSFs with 2 *SLC7A9* variants had highly elevated urinary excretion of dibasic amino acids, supporting the presence of the variants in *trans*, while the third one did not (SKSC_05_0066: *SLC7A9* variants p.(Ala354Thr) and p.(Ala182Thr); Supplemental Table 2). No NKSF had 2 LP/P variants in the same gene. One male KSF was hemizygous for an LP variant in *OCRL*.

#### Dominant KSD.

Monoallelic LP/P variants in AD or AD/AR genes (genes previously ascribed both recessive and dominant inheritance modes) were present in 8.1% (*n* = 57) of KSFs, most commonly in *SLC7A9* (*n* = 9, 1.3%), *SLC34A3* (*n* = 9, 1.3%), *SLC9A3R1* (*n* = 6, 0.86%), *CYP24A1* (*n* = 8, 1.1%), *ALPL* (*n* = 8, 1.2%), or *SLC4A1* (*n* = 4, 0.6%). Five KSFs carried a monoallelic variant in a second gene (*SLC9A3R1*, *BSND*, *SLC3A1*, *FAM20A*) in addition to biallelic LP/P variants in an AR gene or monoallelic LP/P variants in an AD/AR gene. Strikingly, we also found monoallelic LP/P variants in AD or AD/AR genes in 12 NKSFs, mostly in *SLC7A9* (*n* = 5, 2.5%) and *SLC9A3R1* (*n* = 3, 1.3%), where it was the same recurrent *SLC9A3R1* variant [p.(Arg153Gln)] that was found in all KSFs and NKSFs and *CYP24A1* (*n* = 2, 1%). Among the participants with *SLC7A9* variants, we also found 1 recurrent variant, p.(Ala182Thr), in 5 KSFs and 1 NKSF, whereas the remaining variants in this gene were not recurrent (Supplemental Table 2).

#### Carriers for recessive KSD.

Single monoallelic LP/P variants in an AR gene were detected in 5.4% (*n* = 38) of KSFs (Figure 1C and Supplemental Table 2) and in 3.5% (*n* = 7) of NKSFs (Figure 1C and Supplemental Table 3).

#### Variants of uncertain significance and benign variant carriers.

In addition to these LP/P variants, we found that 35.6% (*n* = 256) of KSFs and 45% (*n* = 90) of NKSFs harbored at least 1 VUS in 1 of the 39 genes. We found that 18.4% (*n* = 143) of KSF participants and 17.5% (*n* = 34) of NKSF participants carried BL/B variants. In 30.8% (*n* = 229) of KSFs and 28.3% (*n* = 56) of NKSFs, no variant was identified in any of the 39 analyzed genes. Taken together, these results show that only approximately one-third of participants, whether presenting with KSs or not, lacked a rare variant in all 39 assessed genes and that a large proportion (35.6% in KSFs and up to 45% in NKSFs) carried a VUS.

LP/P variants are slightly more frequent in KSFs than in NKSFs, and up to 9.8% of KSFs could be considered to present with monogenic KSD based on previously reported variants and a previously ascribed inheritance mode for the corresponding gene (*n* = 69/701 [9.8% = 1.6% (presumed) biallelic variants in AR genes + 3.4% monoallelic variants in AD genes + 4.7% monoallelic variants in AR/AD genes + 0.1% X-linked recessive]; Figure 1C). Note, however, that using the same definition, up to 7% of NKSFs (*n* = 10/200 [7% = 3.5% monoallelic variants in AD genes + 3.5% monoallelic variants in AR/AD gene]; Figure 1C) would also receive a diagnosis of monogenic KSD, without having developed KSs.

While the individuals with biallelic LP/P variants are indeed likely to be considered by most clinicians as falling under a monogenic KSD diagnosis, the situation is less clear for participants harboring only monoallelic LP/P variants, in particular in *SLC34A1*, *SLC9A3R1*, *CYP24A1*, or *SLC7A9*, where NKSFs have a rate of variants similar to that of KSFs in these cohorts. To clarify the clinical significance of such variants, we decided to take advantage of the detailed biochemical data available, focusing on genes associated with selected calcium- and phosphate-handling or cystinuria, which are most commonly affected in these questionable situations.

### Correlating biochemical values to genetic findings

#### Phosphate- and calcium-handling genes.

Phosphate-handling genes playing a key role in controlling urinary phosphate excretion include the renal phosphate transporters NaPi-IIA (*SLC34A1*), NaPi-IIC (*SLC34A3*), and their regulator NHERF-1 (*SLC9A3R1*) (8). While only monoallelic *SLC9A3R1* variants have been suggested to cause KSD, both mono- and biallelic *SLC34A1* and *SLC34A3* variants have been implicated in KSD (30–35). Similarly, for *CYP24A1*, which is involved in calcium homeostasis, biallelic pathogenic variants cause infantile hypercalcemia, whereas monoallelic variant carriers may present with a milder phenotype and an increased risk for KSD (27).

We identified monoallelic LP/P variants in *SLC34A1* in 3 KSFs and 2 NKSFs, as well as variants in *SLC9A3R1* in 6 KSFs and in 3 NKSFs, while LP/P *SLC34A3* variants were only identified in KSFs (*n* = 9). No participant in either group had 2 LP/P variants in any of these genes. LB/B and VUS variants were identified in similar proportions in KSF and NKSF participants. For *CYP24A1*, we identified 2 KSFs with biallelic (homozygous) LP/P variants and 8 KSFs with monoallelic LP/P variants, whereas 2 NKSFs had monoallelic LP/P variants (and none had biallelic variants).

We next analyzed the biochemical profile of these individuals to determine whether the presence of monoallelic variants in phosphate-handling transporters/regulators was associated with altered biochemical parameters. We compared phosphate- and calcium-related parameters in KSFs at visit 2 (2 weeks after enrollment) and NKSFs carrying monoallelic variants in *SLC34A1*, *SLC34A3*, and *SLC9A3R1*, as well as *CYP24A1*, with those of a KSF control group without LP/P variants in any of these 3 or other KSD genes. Our phenotyping included the tubular threshold for phosphate reabsorption (tubular maximum reabsorption of phosphate/glomerular filtration rate [TmP/GFR]), plasma phosphate levels, the phosphate-regulating hormones PTH, FGF23 and 1,25-(OH)_2_ vitamin D_3_, the urinary calcium to creatinine ratio, and the EQUIL2-based urinary supersaturation scores for calcium oxalate and brushite (calcium phosphate) stones.

#### SLC34A1.

**Figure 2:**
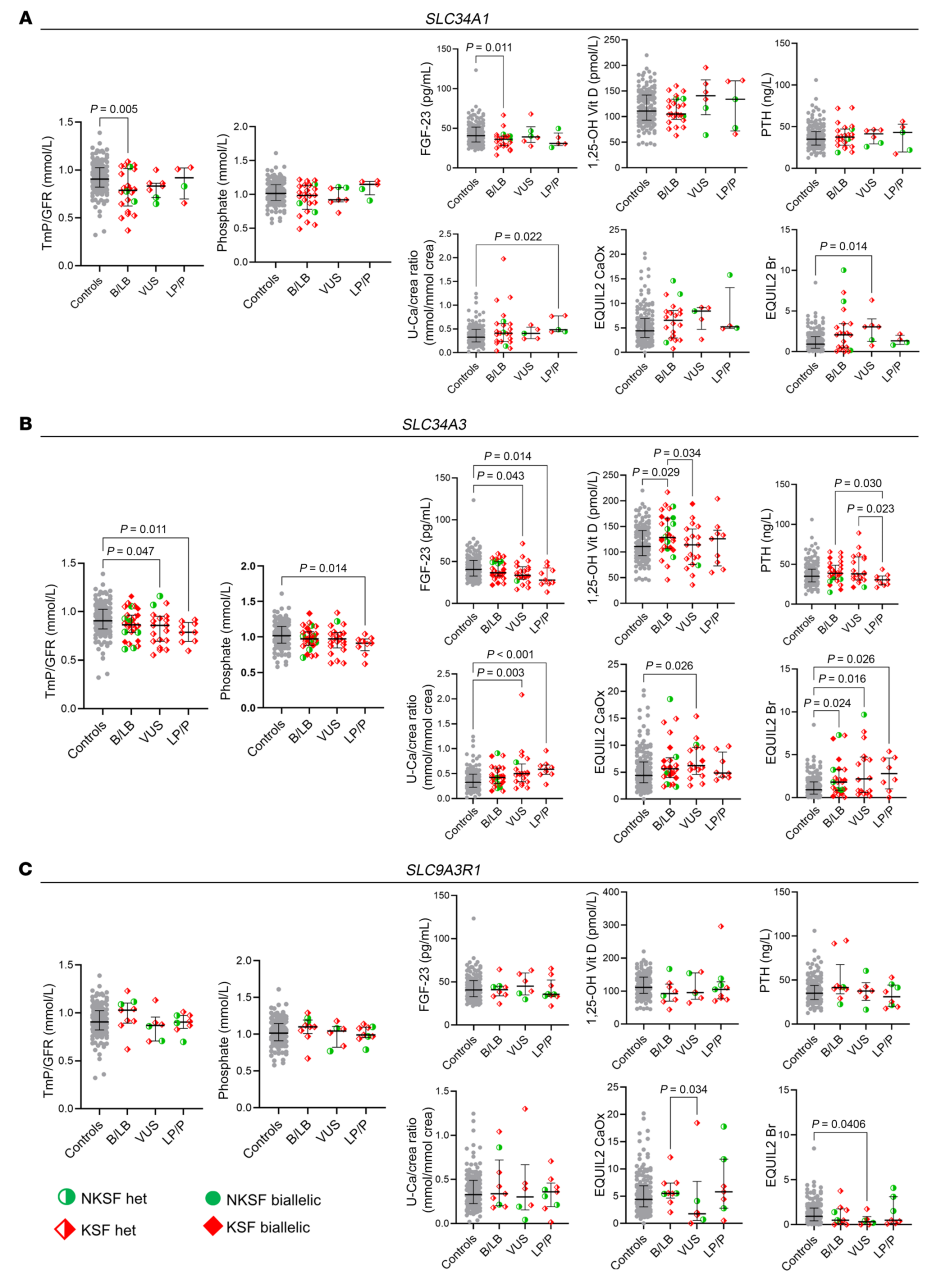
Biochemical effects of heterozygous (monoallelic) *SLC34A1*, *SLC34A3*, and *SLC9A3R1* variants. Biochemical analysis of urine and blood in KSFs and NKSFs shown with the median and IQR, grouped according to the ACMG/AMP class of their *SLC34A1* (**A**), *SLC34A3* (**B**), and *SLC9A3R1* (**C**) variants. Red diamond symbols indicate KSFs, and green circle symbols indicate NKSFs. Filled symbols indicate (presumed) biallelic variants, and half-filled symbols indicate monoallelic variants. Analyzed parameters include the tubular threshold for phosphate reabsorption (TmP/GFR), plasma phosphate levels, the phosphate regulating hormones FGF23, 1,25-(OH)2 vitamin D3 and PTH, and the urinary calcium to creatinine ratio, the EQUIL2-based urinary supersaturation scores for calcium oxalate and brushite. For display clarity, only statistically significant differences (*P* < 0.05) between groups are indicated, as determined by Kruskal-Wallis statistical test. Br, brushite; CaOx, calcium oxalate; U-Ca, urinary calcium; crea, creatinine.

All KSFs with LP/P *SLC34A1* variants had mixed calcium oxalate– and calcium phosphate–containing stones (Supplemental Table 2). Age at first KS was not significantly different between individuals with *SLC34A1* variants compared with controls (Supplemental Figure 5). Renal phosphate excretion showed no significant differences between *SLC34A1* variant carriers and controls independently of the ACMG variant classification (except for lower TmP/GFR and FGF23 in L/B variant carriers; Figure 2A). Plasma phosphate, 1,25-(OH)_2_ vitamin D_3_, and PTH revealed no significant difference across groups, and median values remained within reference ranges. Only urinary Ca/Crea ratios were slightly increased in individuals with LP/P *SLC34A1* variants, but EQUIL2 scores for calcium oxalate and calcium phosphate (brushite) were not significantly different for these individuals. Collectively, these findings suggest that monoallelic LP/P *SLC34A1* variants have at best a minor effect on renal solute excretion and metabolic KSD risk. However, given the small sample sizes, we cannot rule out the possibility that this effect could become statistically significant with larger datasets, which are unfortunately challenging to collect.

#### SLC34A3.

KSFs with LP/P *SLC34A3* variants had calcium oxalate/calcium phosphate stones. Age at first KS event tended to be lower in KSFs with monoallelic LP/P *SLC34A3* variants, but this did not reach statistical significance (Supplemental Figure 5). Compared with the KSF controls who did not harbor variants in phosphate-handling genes, KSFs carrying heterozygous *SLC34A3* LP/P variants had a significantly lower TmP/GFR and also showed reduced plasma phosphate levels. LP/P (and VUS) carriers had higher urinary calcium levels, and their EQUIL2 scores for brushite were elevated (Figure 2B). Thus, the presence of monoallelic LP/P *SLC34A3* variants was associated with the expected biochemical alterations including renal phosphate wasting with reduced plasma phosphate levels and increased urinary calcium excretion.

#### SLC9A3R1.

Stone composition was more variable in *SLC9A3R1* LP/P variant carriers, also including uric acid stones (Supplemental Table 2). Age at first KS event was not significantly different between individuals with *SLC9A3R1* variants compared with controls (Supplemental Figure 5). All but 1 individual (KSFs and NKSFs) harbored the same variant p.(Arg153Gln) in *SLC9A3R1* (Supplemental Table 2), which has been previously classified as pathogenic (36–38). The TmP/GFR ratio of these LP/P variant carriers was not reduced when compared with controls or carriers of variants classified as LB/B or VUS. Likewise, urinary supersaturation (EQUIL2) was similar in all (Figure 2C), except for VUS carriers, who had lower values than LB/B and controls for calcium oxalate and brushite. Given the absence of a biochemical phenotype in individuals with LP/P *SLC9A3R1* variants, and the higher prevalence of *SLC9A3R1* variants in NKSF (Supplemental Tables 2–4), our findings do not support monoallelic variants in this gene [or at least for this particular variant p.(Arg153Gln)] as a strong risk factor for KSD.

#### CYP24A1.

All KSFs harboring biallelic or monoallelic *CYP24A1* LP/P variants displayed calcium oxalate/calcium phosphate stones (Supplemental Table 2). Monoallelic LP/P variant carriers did not display significantly altered biochemical parameters in the urine or blood, except for slightly decreased PTH levels, while 1,25-(OH)_2_ vitamin D3 levels were unaffected (Supplemental Figure 6). The 2 biallelic LP/P variant carriers of *CYP24A1* had the expected biochemical alterations, but given the small number of individuals, this did not reach statistical significance. Our findings do not support a strong effect of monoallelic *CYP24A1* LP/P variants in causing KSD.

### Cystinuria genes

*SLC3A1* and *SLC7A9* encode the essential subunits of a renal amino acid transporter that mediates the reabsorption of cystine and dibasic amino acids. While biallelic *SLC3A1* or *SLC7A9* variants clearly cause monogenic KSD in a recessive inheritance mode, only monoallelic *SLC7A9* variants have been considered sufficient to cause disease in a dominant mode.

*SLC3A1* biallelic LP/P variants were detected in 5 KSFs (3 homozygous, 1 proven compound heterozygous, 1 presumed compound heterozygous), whereas 15 KSFs harbored heterozygous LP/P variants and 18 heterozygous VUS. In comparison, 2 NKSFs had monoallelic LP/P variants in *SLC3A1* (Supplemental Table 3).

**Figure 3:**
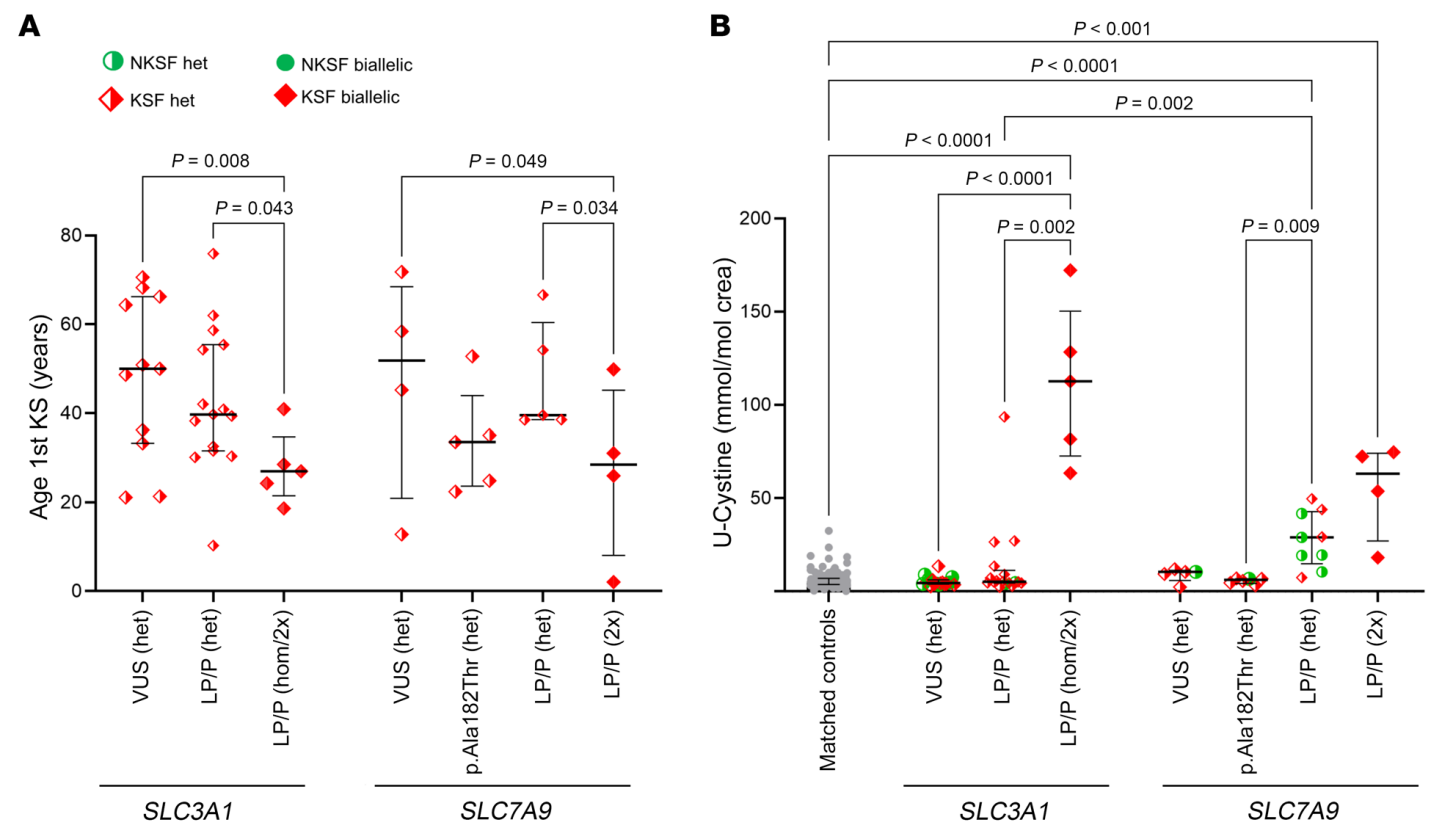
Biochemical alterations in bi- and monoallelic *SLC3A1* and *SLC7A9* variant carriers. Biochemical and clinical characteristics of KSFs and NKSFs according to the ACMG/AMP class of their *SLC3A1* and *SLC7A9* variant. Red diamond symbols indicate KSFs, and green circle symbols indicate NKSFs. Filled symbols indicate (presumed) biallelic variant, while half-filled symbols indicate monoallelic variants. (**A**) Age at first KS event for KSFs with *SLC3A1* or *SLC7A9* monoallelic and presumed biallelic variants. (**B**) Urinary cystine to creatinine ratio in KSFs and NKSFs with single or presumed biallelic variants in *SLC3A1* or *SLC7A9* compared with an age- and sex-matched control group consisting of KSFs and NKSFs without *SLC3A1* or *SLC7A9* variants. Individuals harboring the SLC7A9 p.(Ala182Thr) in a monoallelic state are plotted separately from other heterozygous SLC7A9 LP/P variant carriers. The median and IQR are shown. For display clarity, only statistically significant differences (*P* < 0.05) between groups are indicated, as determined by Kruskal-Wallis statistical test. 2x, individuals with 2 variants in the same gene (proven or presumed biallelic); het, heterozygous; hom, homozygous; U-Cystine, urinary cystine.

We found 4 KSFs with presumed biallelic LP/P *SLC7A9* variants (2 confirmed compound heterozygous, 2 presumed compound heterozygous), and 9 (1.3%) KSFs with monoallelic LP/P variants. Monoallelic *SLC7A9* LP/P variants were also detected in 5 (2.5%) NKSFs, but no NKSF had biallelic LP/P variants in this gene. The previously described *SLC7A9* p.(Ala182Thr) variant was found in both KSFs and NKSFs and appeared to be enriched in our local cohort compared with gnomAD data (Supplemental Table 4). These findings are consistent with the fact that the presence of biallelic LP/P variants in *SLC3A1* or *SLC7A9* causes KSD. As expected, the age at the first KS event was significantly lower in KSFs with biallelic LP/P variants in *SLC3A1* or *SLC7A9* (Figure 3A).

In contrast, the high prevalence of monoallelic *SLC7A9* LP/P variants in NKSFs raises the question of their clinical significance. We therefore next analyzed the type of KSs found in participants with biallelic and monoallelic LP/P variants in these genes. Cystine stones were found in all KSFs with biallelic *SLC3A1* LP/P variants and in all but 1 *SLC7A9* biallelic-LP/P variant carriers (who presented earlier with a calcium oxalate stone and later with a cystine stone). In contrast, KSFs with monoallelic LP/P *SLC7A9* variants all presented with calcium oxalate stones.

For comparison of the biochemical phenotype of both KSFs and NKSFs harboring variants in the 2 cystinuria genes, we selected an age- and sex-matched KSF control group without variants in *SLC3A1* and *SLC7A9*. The urinary concentrations of cystine, ornithine, lysine, and arginine were measured in affected KSFs with LP/P variants in these 2 genes and in KSFs without such variants, who were considered matched controls (Supplemental Table 5). Consistent with the observation from the stone analysis, KSFs harboring biallelic *SLC3A1* LP/P variants had significantly increased urinary cystine concentrations, along with elevated concentrations of the dibasic amino acids ornithine, lysine, and arginine (Figure 3B and Supplemental Tables 5 and 6). In contrast, mean cystine excretion was not significantly increased in KSFs or NKSFs with monoallelic *SLC3A1* LP/P variants, although 2 KSFs had borderline elevated cystinuria levels (with calcium oxalate stones), and 1 KSF had substantially elevated urinary cystine levels as well as cystine stones (Supplemental Tables 2 and 5). This individual appears to be an outlier compared with the other 14 KSFs with monoallelic variants who had normal cystine excretion (and non-cystine stones), raising the suspicion that a second variant in *SLC3A1* (or *SLC7A9*) might have been missed with WES, possibly because it was a noncoding variant.

KSFs with presumed biallelic LP/P variants in SLC7A9 had significantly higher urinary cystine levels than did controls (*P* < 0.001, Figure 3 and Supplemental Table 5). Monoallelic carriers of SLC7A9 LP/P variants had moderately but significantly elevated urinary cystine levels, in both KSFs and NKSFs (*P* < 0.0001, Figure 3 and Supplemental Table 5). In contrast, carriers of the recurrent p.(Ala182Thr) variant in a monoallelic state had normal urinary cystine levels, as did carriers of VUS in either gene.

Taken together, analysis of the biochemical phenotype and comparison with NKSFs confirmed that only biallelic LP/P variants in *SLC3A1* and *SLC7A9* caused strong biochemical abnormalities resulting in monogenic KSD with cystine stones, whereas *SLC7A9* monoallelic variants moderately increased urinary cystine levels, except for the p.(Ala182Thr) variant in *SLC7A9*, for which no biochemical effect could be found.

### The presence of high-risk variants in KS genes correlates with increased KS recurrence

**Figure 4:**
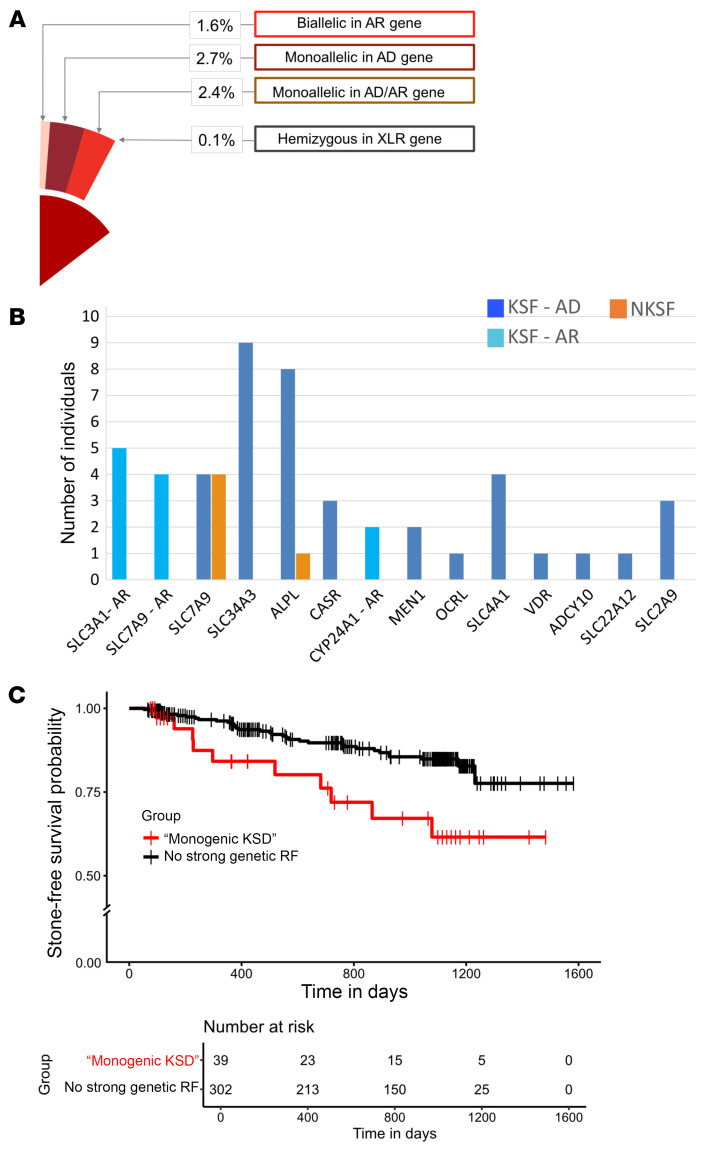
The presence of LP/P variants in KSD genes is associated with higher KS recurrence. (**A**) Reclassification of the clinical significance of monoallelic variants in *CYP24A1*, *SLC9A3R1*, and *SLC34A1* and of the p.(Ala182Thr) *SLC7A9* variant based on a lack of biochemical effects leads to a “solve rate” of 6.8% of monogenic KSD in this cohort. The indicated wedge refers to the pie chart in Figure 1C for KSFd (15.2% of KSFd with LP/P variants) to illustrate how reclassification of these variants modifies the proportions of KSFs with monogenic KSD. (**B**) Bar plot indicating the final monogenic KSD diagnoses in this cohort. (**C**) Kaplan-Meier curve showing the time to KS recurrence in KSFs with monogenic KSD and in KSFs without such strong genetic risk factors (RF) (*P*_log-rank_ = 0.0006). Of note, this difference remained significant when sex and age were used as covariates (*P*_Cox proportional hazards regression_ = 0.015). The figure was created using the R package “survminer” (versiom 0.5.0). AR, autosomal recessive; XLR, X-linked recessive.

Correlation of the detailed biochemical analyses performed with the genetic variants identified and comparison of KSFs with NKSFs led us to reclassify heterozygous variants in *SLC9A3R1*, *SLC34A1*, and *CYP24A1* as well as the common p.(Ala182Thr) variant in *SLC7A9* — all previously classified as LP/P according to ACMG/AMP criteria and supposedly disease-causing in the monoallelic state — as not sufficient to explain KSD in a monogenic model alone. After excluding these variants, we found that 6.8% (*n* = 48) of the KSFs studied here harbored a strong genetic risk factor for KSD in the form of a LP/P variant in a known KSD gene (Figure 4, A and B).

We next sought to determine the clinical relevance of such strong genetic risk factors for predicting clinical outcomes. Taking advantage of the 3-year follow-up period we had for KSFs, we compared the recurrence rate for KSs between KSFs with high-risk genetic variants and those without such strong genetic risk factors. We found a significant difference in time to first KS recurrence between KSFs with strong genetic risk factors and those without such strong risk factors (log-rank *P* = 0.0006; Figure 4C). Of note, this difference remained significant after adjusting for sex and age as covariates in a Cox proportional hazards regression model (HR [95% CI] = 2.4506 [1.1939 – 5.030], *P* = 0.015) (Supplemental Table 7). Furthermore, 5 of 48 (10.4%) KSFs with strong genetic risk factors experienced more than 1 recurrence during the follow-up period, compared with 7 of 302 (2.3%) KSFs without such strong genetic risk factor. KS composition varied between 2 stone episodes for 30 of 185 individuals, including for carriers of KSD-causing genetic variants (Supplemental Figure 7).

## Discussion

KSD eventually results from the urinary imbalance of crystallization promoters and inhibitors. In the complex interplay of genetic and environmental factors underlying KSD, interpreting the significance of genetic variants in KSD genes identified in adults with KSD remains challenging. Not only is it difficult to classify individual variants as pathogenic or not, but it also remains unclear to what extent monoallelic variants in some KSD genes affect the risk of developing KSD. By combining comprehensive genetic analyses with extensive biochemical phenotyping in an adult cohort of KSD and through comparison with a NKSF cohort, we clarify the clinical significance of variants in known KSD genes. We further show that the presence of bona fide LP/P variants in KSD genes is associated with a higher KS recurrence rate. This thorough systematic analysis illustrates how detailed genotype-phenotype correlation analysis improves the interpretation of the clinical significance of genetic variants.

Our findings suggest that several previously described variants in KSD genes are unlikely to represent strong enough risk factors to be causal for monogenic KSD in the proposed inheritance modes. For instance, we found that heterozygous (monoallelic) variants in *CYP24A1*, *SLC34A1*, or *SLC9A3R1* had a minimal effect on urine or blood biochemistry and were not enriched in KSFs compared with NKSFs. In contrast, our study confirms that single heterozygous LP/P variants in *SLC34A3* or *SLC7A9* significantly affected urinary biochemistry toward promoting crystallization, albeit with an intermediate effect size compared with biallelic variants in the same genes, consistent with recent reports from kindreds and large cohorts of carriers of *SLC34A3* variants (35, 39). Interestingly, we also observed monoallelic variants in some KSD genes with relatively high frequency in NKSFs, indicating that heterozygous LP/P variants in *SLC7A9*, for example, may require additional factors for the development of KSD and should be considered as intermediate/strong risk factors for KSD rather than true monogenic KSD.

Biallelic *SLC34A3* variants cause hereditary hypophosphatemic rickets with hypercalciuria in an autosomal recessive mode (31). Heterozygous carriers may have a higher rate of renal calcifications and lower phosphate levels than noncarriers (32, 35). Within our cohort, monoallelic pathogenic *SLC34A3* variants were detected exclusively in KSFs and were associated with hyperphosphaturia, hypophosphatemia, and hypercalciuria, as well as elevated urinary brushite (calcium phosphate) supersaturation. This suggests that monoallelic *SLC34A3* carriers may present with an intermediate phenotype consistent with a dosage effect in which monoallelic variants in recessive genes induce a milder phenotype (11). This finding is supported by recent analyses from the 100,000 Genomes Project, in which exome-wide enrichment of rare deleterious monoallelic *SLC34A3* variants was found to be the most important risk factor for KSD (28).

Furthermore, our results confirm that cystinuria is caused by biallelic SLC3A1 and SLC7A9 variants in a recessive mode, whereas heterozygous SLC7A9 variants confer an increased risk for kidney stones in a dominant mode with incomplete penetrance. As expected, biallelic SLC3A1 LP/P variant KSF carriers had the highest urinary cystine excretion, followed by biallelic SLC7A9 LP/P variant KSF carriers. In heterozygous *SLC7A9* LP/P variant carriers, urinary cystine was increased in both KSFs and NKSFs. Notably, our findings show that monoallelic carriers of the allegedly pathogenic variant p.(Ala182Thr) in *SLC7A9* (40, 41) had normal urinary cystine excretion. This variant did, however, contribute to cystinuria when in *trans* with a loss-of-function allele in at least 1 individual and is therefore probably a hypomorphic variant.

Our assessment of urinary cystine in a large group of KSFs provides valuable new insights. This unbiased approach identified adult patients with a new diagnosis of cystinuria. Interestingly, monoallelic *SLC7A9* variants were associated with only moderately elevated urinary cystine and were typically associated with noncystinuric KSs. However, our results suggest that these carriers still have an increased risk of stone formation and highlight the importance of considering heterozygous *SLC7A9* variants as a significant risk factor for KSD, even in the absence of classic cystinuria. It also emphasizes the value of urinary cystine measurement in broader KSD populations, regardless of cystine stone composition, as it may allow for the detection of heterozygous *SLC7A9* variant carriers.

After reinterpretation of the clinical significance of genetic variants in KSD genes based on biochemistry and on comparison with a control cohort of NKSFs, we identified a strong genetic component for KS development in 6.8% of KSFs. This percentage is lower than in selected cohorts focusing on very early disease onset but consistent with results of other relatively unselected adult KSF cohorts, in which a diagnostic yield of approximately 2.7%–8% was reported (17–20, 22, 23). This reclassification of variants is relevant, as the presence of bona fide LP/P variants representing strong genetic risk factors correlated with a higher risk for KS recurrence than in individuals not harboring such variants. Given the described association between KSD and the increased risk for CKD (42, 43), recurrent stones may have an even more deleterious effect on kidney function. Whether the presence of genetic variants with strong effects was associated with a more rapid decrease in the estimated GFR (eGFR) could not, unfortunately, be tested in our cohort, given the small sample sizes resulting in insufficient statistical power.

Our study has some limitations. First, despite being among the largest reported cohorts of uniformly recruited individuals with KSD, the numbers are nonetheless small, thus limiting statistical analyses. This might explain, for example, the fact that we did not observe a statistically significant effect on biochemical parameters in carriers of heterozygous *SLC34A1* variants, in contrast to a recent study focusing on *SLC34A1* (and *SLC34A3*) that included a larger number of carriers (35). In addition, even in those instances in which we observed statistically significant differences, we did not correct for multiple testing (given the small number of cases and the number of comparisons performed). However, since several biochemical parameters for the genes tested showed consistent findings expected with dysfunction of the respective protein, this increases the confidence in the interpretation of the biological significance of the tested variants for *SLC34A3* and *SLC7A9*. Moreover, while every effort was made to have complete datasets with all parameters at each visit, the follow-up for some participants was incomplete (i.e., visits were complicated by the COVID-19 pandemic), which led to exclusion of some participants for some analyses due to a lack of data. Thus, the power of the analyses was limited for most genes by the rarity of the variants identified, which is a challenge inherent to the study of most genetic diseases. Given the typical challenges of participant enrollment, the control group (NKSFs) was also not perfectly matched to the cases (KSFs) with respect to sex and age, which may have introduced some biases. However, the biochemical parameters were mostly compared here between KSFs with and without genetic variants, such that those comparisons were not subject to this limitation. Another limitation was the lack of adjustment for concomitant medications. This observational cohort was designed to provide a comprehensive and longitudinal follow-up of adults with KSs, rather than intervention. Therefore, drugs were neither initiated nor withheld for study purposes. A subset of participants reported taking various medications, some of which could have affected the biochemical analyses. However, despite this, we observed significant correlations between the presence of some genetic variants and the biochemical alterations expected from dysfunction of the corresponding transporter protein, supporting a variant-driven effect, which is the conclusion we draw from this work. Finally, the cohort consisted almost completely of individuals of European ancestry, which may limit the generalizability of the findings. However, as the local population is highly mixed within the European context, we do not expect founder effects or similar population-specific biases, and, indeed, only a few variants occurred in more than 1 individual.

Our study also has several major strengths. This work presents an unbiased genetic analysis of a large cohort of adults with KSD combined with detailed biochemical analysis allowing for assessment of genotype-phenotype correlations. Moreover, longitudinal observations enable correlations with outcomes such as KS recurrence. By integrating genetic and biochemical phenotyping, our study improves the classification of variants in individuals with KSD and identifies those at higher risk for recurrent stone events. We were also able to identify previously undiagnosed adult patients with cystinuria and clarify the relevance of frequent variants such as those in *SLC7A9*. Altogether, integrating genetic data with detailed biochemical analyses improves the interpretation of the clinical relevance of genetic findings. Combined with the consideration of acquired risk factors (e.g., hypertension, diabetes, dyslipidemia), this personalized diagnostic approach holds promise for improving outcomes for patients with recurrent KSD and for guiding tailored monitoring and therapeutic strategies.

## Methods

### Sex as a biological variable.

Individuals of both sexes were included in the KSF and in NKSF groups (Table 1). Correction for sex was performed for the analysis of the recurrence rate of stones (Figure 4C).

### Study cohort.

The SKSC is a multicenter, longitudinal observational study encompassing adult KSFs and NKSFs (29). KSFs include individuals recruited at 6 study sites with a history of more than 1 stone event or a single event coupled with at least 1 additional risk factor (inclusion criteria Supplemental Figure 1). Please note that 80 individuals were also enrolled in the Bern Kidney Stone registry; however, genetic findings have been reported only for 2 of these individuals in Anderegg et al. (23) (SKSC_02_0127 and SKSC_02_0004, see Supplemental Table 2). The NKSFs are individuals recruited from non-nephrology departments at the recruiting centers, had no KSs or a history thereof and no nephrology referral or kidney disease. Absence of stones or nephrocalcinosis in NKSFs was confirmed by a low-dose CT scan. For deep phenotyping, blood and 24-hour urine samples from KSF participants were collected and analyzed repeatedly over a 3-year follow-up period (Figure 1). Urine and blood samples from NKSF participants were obtained at enrollment. To assess urinary supersaturation for calcium oxalate and calcium phosphate, EQUIL2 scores were calculated from 24-hour urine samples (44).

### WES, variant filtering, and classification.

WES was performed on 701 KSF and 200 NKSF participants with subsequent analysis of a virtual panel consisting of 39 established KS genes (Supplemental Table 1). DNA was extracted from peripheral blood, and WES was performed using the IDT xGen Exome Research Panel version 2 (Integrated DNA Technologies) on the NovaSeq 6000 system (Illumina). Alignment and variant calling were done using DRAGEN Bio-IT Platform version 3.9 (Illumina). In order to exclude low-quality variants, variants with a read frequency of less than 0.3 in more than 60% of individuals or with a quality score below 25 were removed from the entire dataset as well as variants occurring in more than 10% of individuals. Average coverage was 202, and exomes with a coverage of less than 100 were repeated. The average duplicate rate was 7.4, the average Q30_bases was 92.72, and the average Ti/Tv was 2.29. Following these quality measures, variants with a GnomAD allele frequency of greater than 0.01 (1%) were excluded (https://gnomad.broadinstitute.org/v2.1.1). Variants present in regions of interest (exons and splice regions up to 10 bp into the intron) that were nonsynonymous were retained (filtering steps in Supplemental Figure 3). Variant classification was performed according to the ACMG/AMP guidelines and ClinGen recommendations for PP3/BP4 criteria (24, 45). Additionally, intronic variants previously described as pathogenic in ClinVar and/or HGMD with sufficient evidence were also retained. If published functional experiments supported the effect of a variant, this was also taken into account (even if the variant was reported as VUS in ClinVar).

### Statistics.

GraphPad Prism 9.5.1 (GraphPad Software) and Python version 3.10 were used for plots, calculations, and statistical analysis. Continuous variables are presented as the median (IQR), since the majority of variables were not normally distributed as determined by a Shapiro-Wilk test. Categorical data are shown as frequencies and percentages. The Mann-Whitney *U* test was applied for comparison of continuous variables between groups and the χ^2^ test for categorical variables. A *P* value of less than 0.05 was considered significant. The respective test used is indicated in the main text and/or figure legend as appropriate. Ancestry was analyzed on the basis of the WES genetic data using the R package EthSEQ (version 3.0.2). The figure for the Kaplan-Meier curve for KS recurrence was created using the R package “survminer” (version 0.5.0). A Cox proportional hazards regression model was used to evaluate the difference between KSFs and NKSFs after adjustment for sex and age as covariates.

### Study approval.

All participants signed an informed consent to participate in this study, which was approved by the Cantonal Ethics Commission of the Canton of Zurich (BASEC 2021-00589).

### Data availability.

All data available, including variant lists, will be shared upon request to the corresponding author (except for per-patient WES data for data protection reasons). Values for all data points in graphs are reported in the Supporting Data Values file.

## Author contributions

JM conceptualized the study, conducted formal analysis and experiments, designed the study methodology, and wrote the original draft of the manuscript. RBG and CAW conceptualized and supervised the study, acquired funding, wrote, reviewed, and edited the manuscript, and handled project administration. JP, JFS, EMC, II, MP, BO, IRA, and PMF performed experiments and formal analysis and designed the study methodology. OB and A Rauch provided resources, supervised the study, and acquired funding. DGF, TE, FB, GW, NAD, A Ritter, SS, HS, and BR handled patient recruitment. All authors reviewed and edited the manuscript.

## Funding support

- National Center for Competence in Research NCCR Kidney. CH, funded by the Swiss National Science Foundation (183774) and the University of Zurich.

## Supplementary Material

Supplemental data

ICMJE disclosure forms

Supporting data values

## Figures and Tables

**Table 1:**
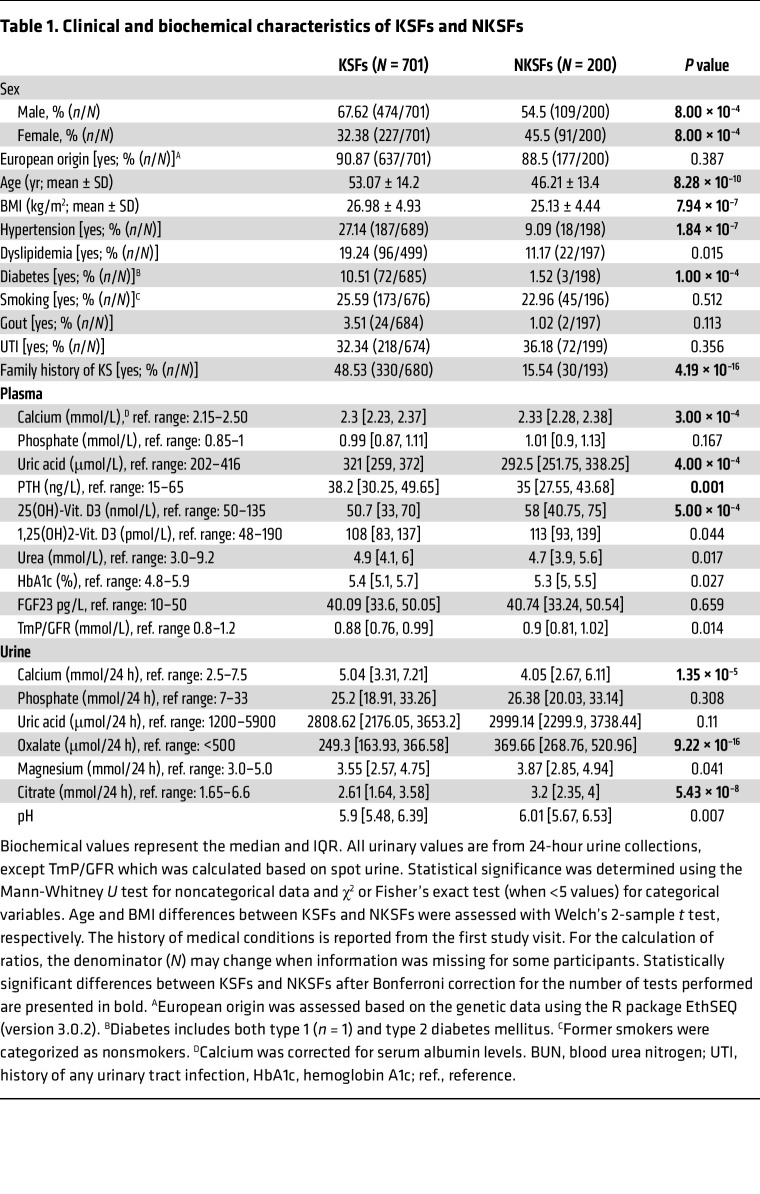
Clinical and biochemical characteristics of KSFs and NKSFs

## Version 1 03/02/2026

Electronic publication
