# Supplementary material for: Deep biochemical phenotyping reveals prognostic value of rare genetic variants in adult kidney stone disease
Source: J Clin Invest. 2026 Mar 2;136(5):e196277. doi: 10.1172/JCI196277 (PMC12948434; doi:10.1172/JCI196277)
Supplement: Supplemental data [file jci-136-196277-s117.pdf]

Supplemental Material:

Deep biochemical phenotyping reveals prognostic value of rare genetic variants in adult kidney stone disease

Johannes Münch,[1,2] Jana Petrovska,[1,3] Joana Figueiro-Silva,[1] Isabel Rubio-Aliaga,[2] Elena M. Cabello,[1] Ivan Ivanovski,[1] Michael Papik,[1] Beatrice Oneda,[1] Daniel G. Fuster,[4,5] Harald Seeger,[6] Thomas Ernandez,[7] Florian Buchkremer,[8] Gregoire Wuerzner,[9] Nasser A. Dhayat,[5] Alexander Ritter,[6] Stephan Segerer,[8] Beat Roth,[10] Anita Rauch,[1] Pietro Manuel Ferraro,[11] Olivier Bonny,[14,9,12] Carsten A. Wagner,[2,4,13] and Ruxandra Bachmann-Gagescu[1,3,13]

[1]Institute of Medical Genetics, University of Zurich, Schlieren-Zurich, Switzerland.
[2]Institute of Physiology, University of Zurich, Schlieren-Zurich, Switzerland.
[3]Department of Molecular Life Sciences, University of Zurich, Zurich, Switzerland.
[4]National Center of Competence in Research (NCCR) Kidney.CH, Bern, Switzerland.
[5]Department of Nephrology and Hypertension, Inselspital, Bern University Hospital, University of Bern, Bern, Switzerland.
[6]Department of Nephrology, University Hospital Zurich, Zurich, Switzerland.
[7]Service of Nephrology, Geneva University Hospitals, Geneva, Switzerland.
[8]Division of Nephrology, Cantonal Hospital Aarau, Aarau, Switzerland.
[9]Service of Nephrology and Hypertension, Lausanne University Hospital and University of Lausanne, Lausanne, Switzerland.
[10]Department of Urology, University Hospital of Bern, Inselspital, University of Bern, Bern, Switzerland.
[11]Section of Nephrology, Department of Medicine, Università degli Studi di Verona, Verona, Italy.
[12]Department of Biomedical Sciences, University of Lausanne, Lausanne, Switzerland.
[13]Zurich Kidney Center, University of Zurich, Zurich, Switzerland.

Table of contents:

Supplemental Figure 1: Detailed inclusion criteria for the study, p.3

Supplemental Figure 2: Composition of kidney stones in the SKSC cohort, p.4-5

Supplemental Figure 3: Filtering pipeline for genetic variants identified by Whole Exome Sequencing (WES), p.6

Supplemental Figure 4: Ancestry analysis of the cohort, p.7

Supplemental Figure 5: Age at first kidney stone occurrence in variant carriers for phosphate-handling genes, p.8

Supplemental Figure 6: Biochemical values for *CYP24A1* heterozygous variant carriers, p.9-10

Supplemental Figure 7: Kidney stone composition in recurrent stone episodes, p.11

Supplemental Table 1: Virtual panel for kidney stone associated genes, p.12

Supplemental Table 2: Clinical and biochemical details for Kidney stone formers harboring (likely) pathogenic variants in a KSD gene, p.13-19.

Supplemental Table 3: (Likely) pathogenic variants identified in non-Kidney Stone formers (NKSF), p.20-21

Supplemental Table 4: LP/P variants recurring more than twice in KSF and/or NKSF, p.22

Supplemental Table 5: Urine concentrations of the amino acids arginine, cystine, lysine and ornithine for KSF and NKSF with (likely) pathogenic variants detected in *SLC3A1* and *SLC7A9* and a double-sized matched control group, p.23

Supplemental Table 6: Genetic variants and urinary dibasic amino acid levels in KSF with cystine stones, p.24

Supplemental Table 7: Hazard ratio for stone recurrence analysis, p.25

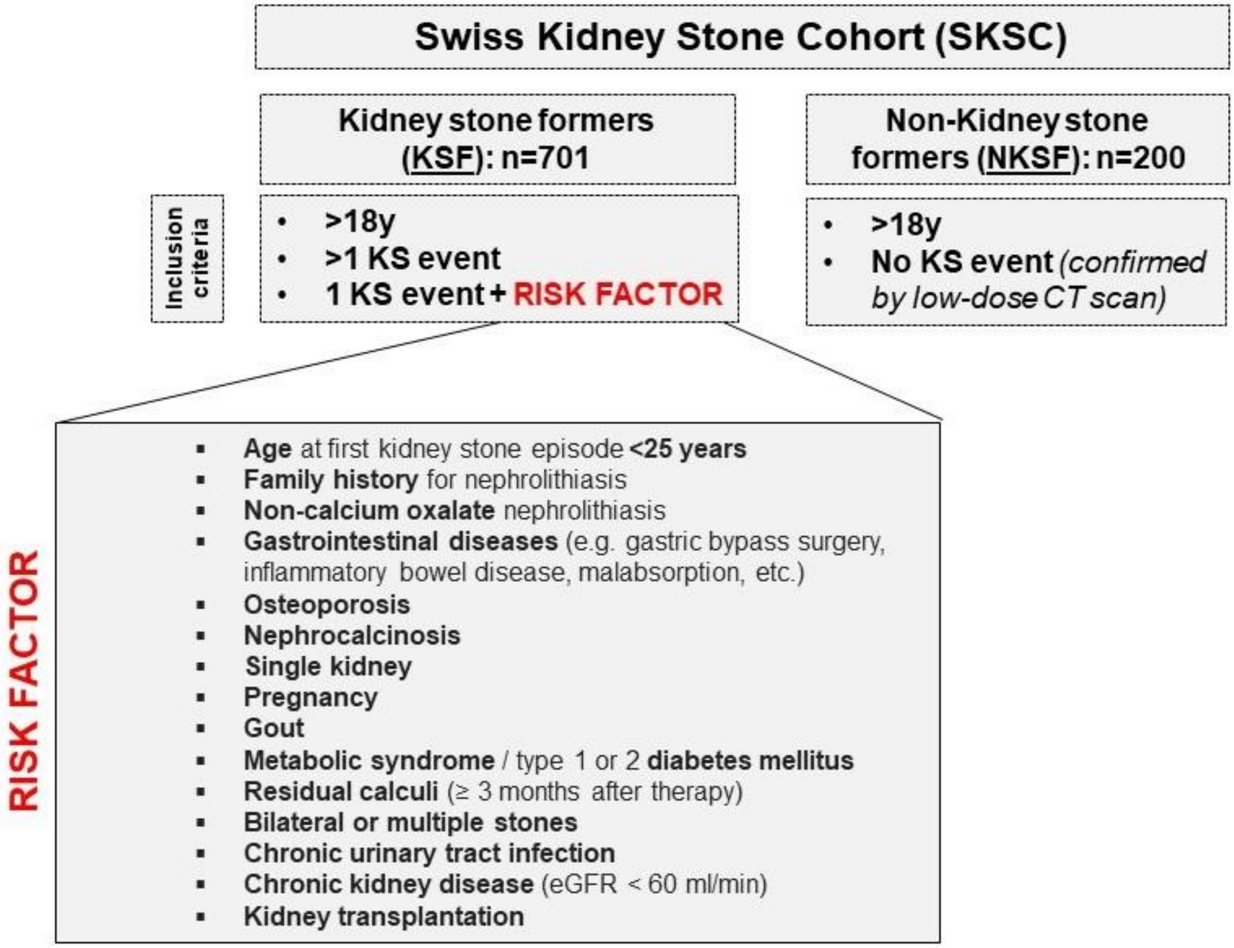

Supplemental Figure 1: Detailed inclusion criteria for the study

The KSF group includes individuals with more than one KS event or a single stone event with at least one additional risk factor. Additional risk factors allowing inclusion in the KSF group with only one reported kidney stone are indicated in the lower box (risk factor). The NKSF group includes individuals with no history of KS and additional confirmation of lack of kidney stone formation by low-dose computed tomography (CT). Abbreviations: CT, computed tomography; eGFR, estimated glomerular filtration rate; KS, kidney stone; KSF, kidney stone former; n, number; NKSF, non-kidney stone former; y, years.

A

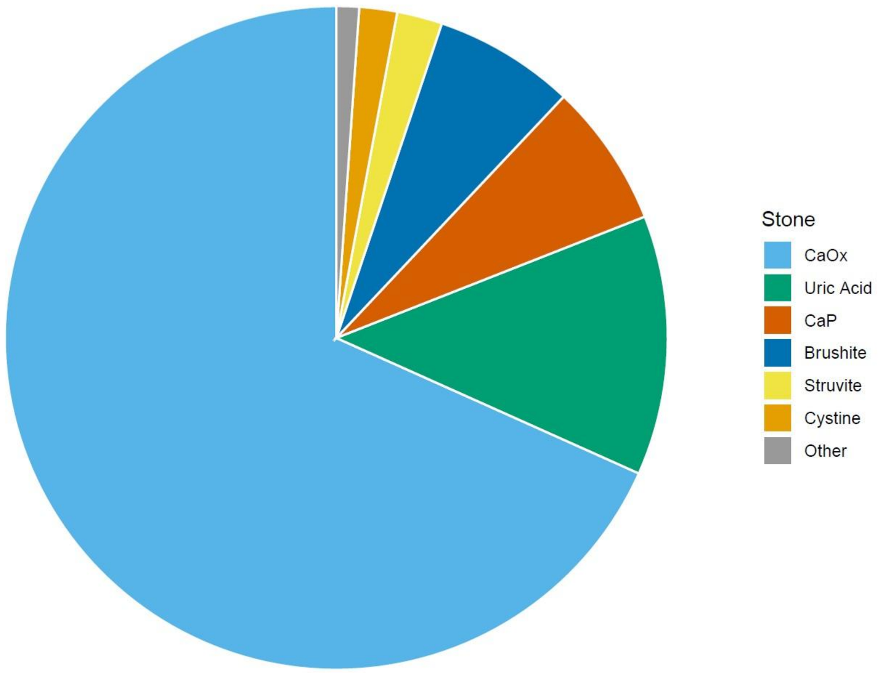

B

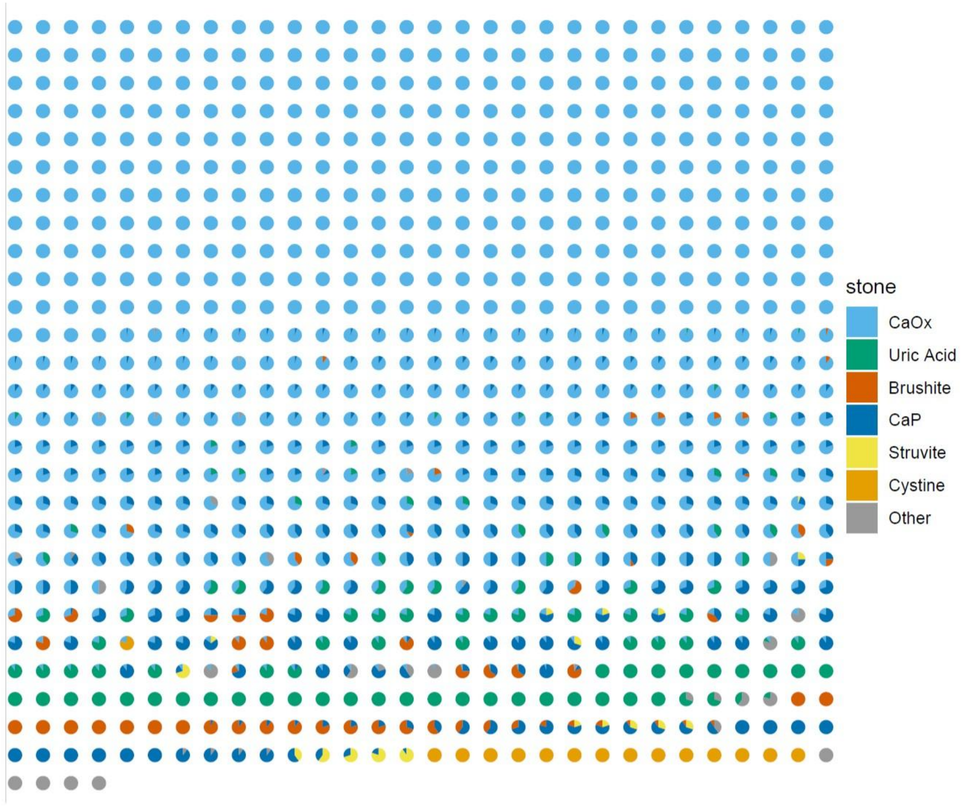

Supplemental Figure 2: Composition of kidney stones in the SKSC

(A) Piechart showing the distribution of kidney stone composition in the SKSC (information based on medical charts). All stones for which the information was available are included in this

analysis, sometimes including multiple stones per participant. Classification of stone composition was performed based on Lieske et al (Lieske et al. 2014): each stone was placed in one unique group: (1) stones containing any struvite (ST) were placed in the ST group; (2) stones containing any cystine (Cy) were placed in the Cy group; (3) stones containing any uric acid (UA) were placed in the UA group; (4) stones containing any brushite (BR) were placed in the BR group; (5) stones were classified as calcium oxalate (CaOx) if they had a majority (>50%) of CaOx with or without any hydroxyapatite (HA); or (6) stones were classified as calcium phosphate (CaP) if they contained a majority (>50%) of Cap (Apatite or Octacalciumphosphate). (7) Other - stones not assigned to any group. Note the clear predominance of calcium oxalate stones. **(B)** Composition of individual stones based on medical record. Each piechart represents a single stone (can include recurrent stones in individual participants). Note that many stones have a mixed composition.

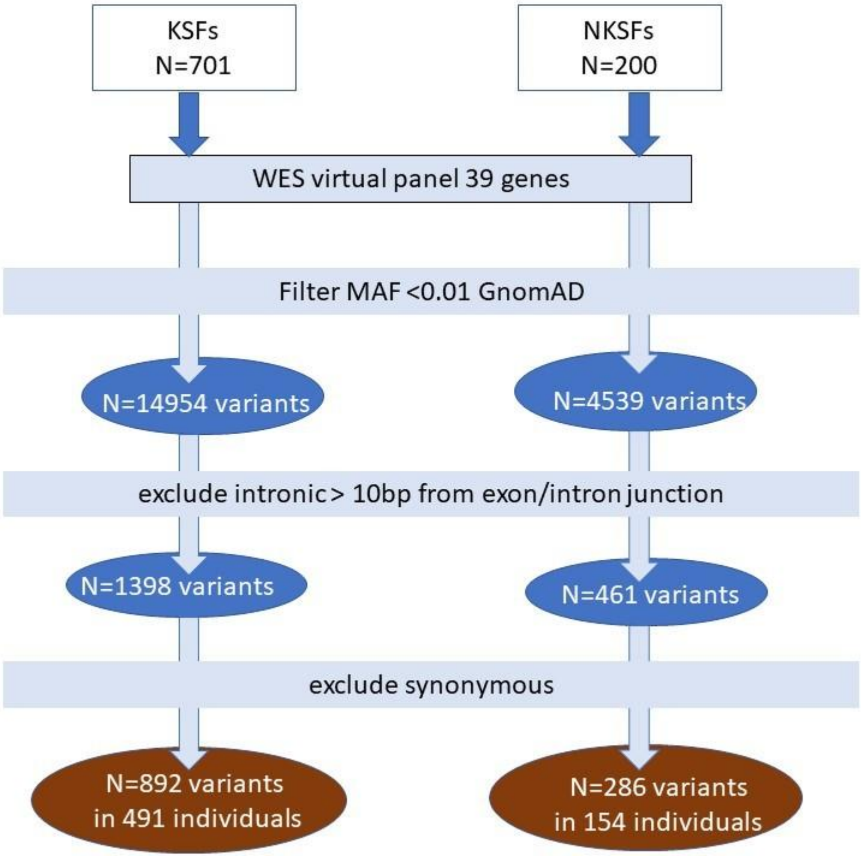

Supplemental Figure 3: Filtering pipeline for genetic variants identified by Whole Exome Sequencing

Whole exome sequencing (WES) was performed on 701 KSF and 200 NKSF participants with subsequent analysis of a virtual panel consisting of 39 established kidney stone genes (**see Supplemental Table S1**). DNA was extracted from peripheral blood and whole exome sequencing (WES) was performed using the IDT xGen Exome Research Panel v2 (Integrated DNA Technologies, Coralville, Iowa, USA) on NovaSeq 6000 system (Illumina Inc.). Alignment and variant calling were done using DRAGEN Bio-IT Platform V3.9 (Illumina Inc.). In order to exclude low quality variants, variants with a read frequency <0.3 in more than 60% of individuals or with a quality score <25 were removed from the entire data set as well as variants occurring in more than 10% of individuals. Following these quality measures, variants with a gnomAD allele frequency >0.01 (1%) were excluded (https://gnomad.broadinstitute.org/ v2.1.1). Variants present in regions of interest (exons and splice regions up to 10 bp into the intron) that were non-synonymous were retained for further classification according to ACMG/AMP criteria. *Abbreviations*: MAF, minor allele frequency; KSF; kidney stone formers; N, number; NKSF, non-kidney stone formers; SKSC, Swiss Kidney Stone Cohort; WES, whole exome sequencing.

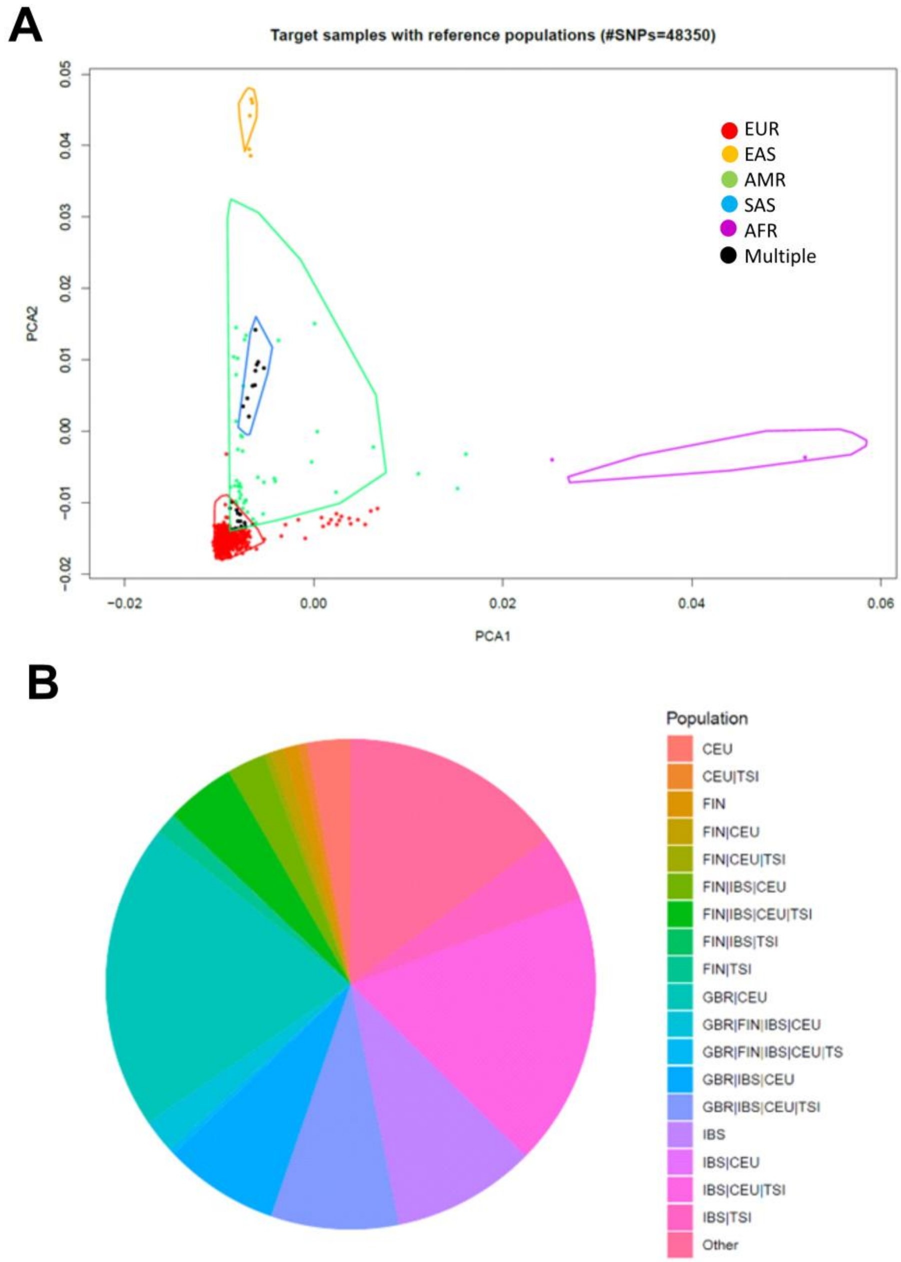

Supplemental Figure 4: Ancestry analysis of the cohort

(A) Ancestry distribution of the SKSC. Ancestry was analyzed based on the genetic data using the R package EthSEQ (v.3.0.2), in 2D space. Abbreviations: AFR, African , AMR, Ad Mixed American, EAS, East Asian, EUR, European, SAS, South Asian. **(B)** Ancestry of participants of European origin was further subdivided into European subpopulations, indicating that this sample is highly mixed within the European context. Abbreviations: CEU (Utah Residents with Northern and Western European Ancestry), TSI (Toscani in) Italia, FIN Finnish, GBR British (England and Scotland), IBS (Iberian population in) Spain.

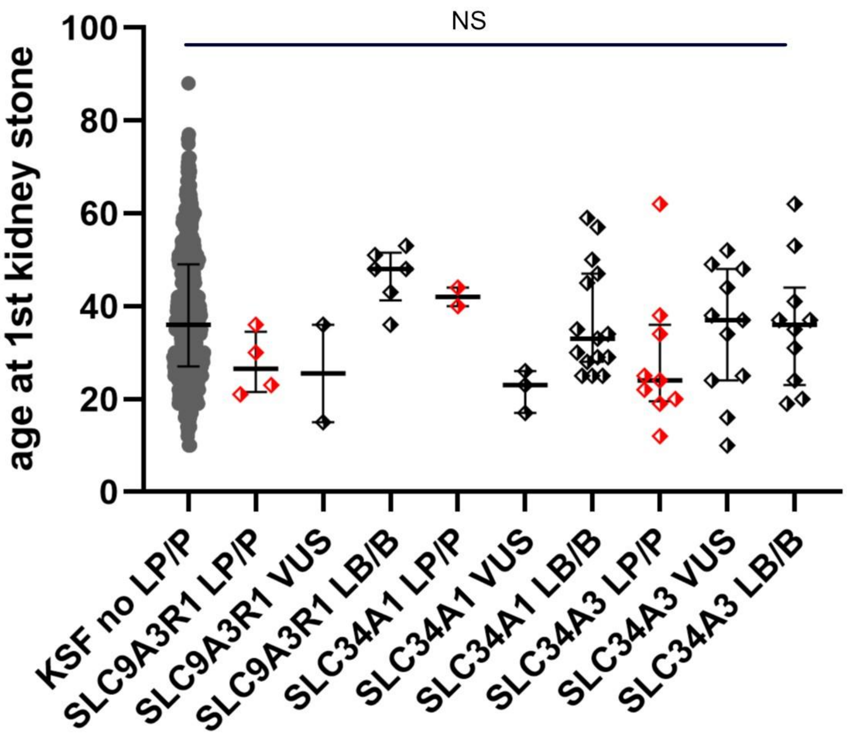

Supplemental Figure 5: Age at first kidney stone occurrence in variant carriers for phosphate-handling genes

Age at first KS event for KSF with variants in *SLC9A3R1, SLC34A1* and *SLC34A3*, compared to KSF without LP/P variants in any of the tested genes. The red symbols indicate individuals with pathogenic or likely pathogenic (LP/P) variants. Datapoints and error bars indicate median and interquartile range. None of the groups shows a significant difference compared to control (Kruskal-Wallis test).

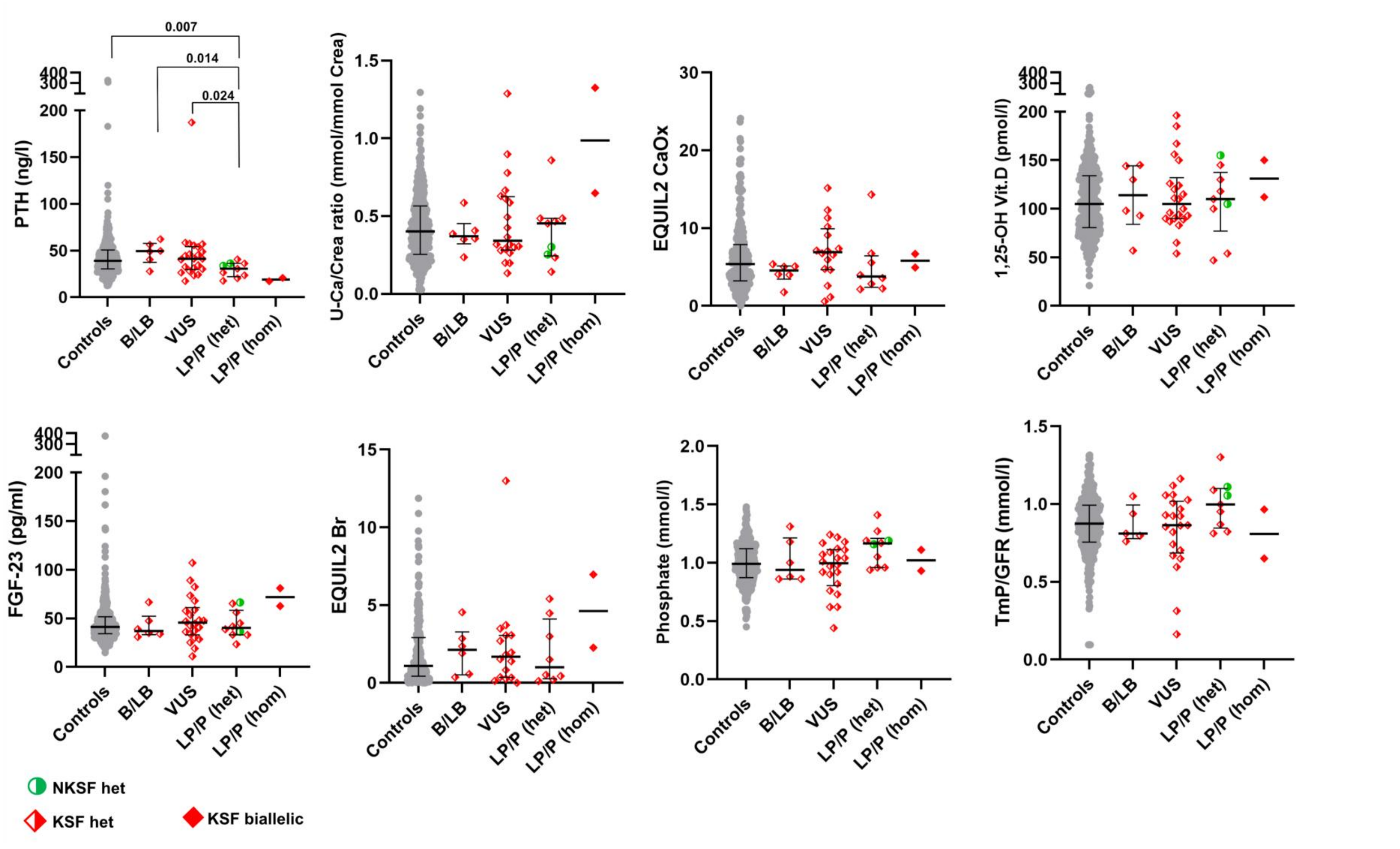

Supplemental Figure 6: Biochemical values for *CYP24A1* heterozygous variant carriers

Biochemical analysis of urine and blood in kidney stone formers (KSF) and non-kidney stone formers (NKSF) grouped according to the ACMG/AMP class of their *CYP24A1* variant. Red diamond symbols indicate KSF and green circle symbols indicate NKSF,

filled symbols indicate biallelic (homozygous- hom) variants while half-filled symbols indicate monoallelic (heterozygous – het) variants. Analyzed parameters include the urinary calcium to creatinine ratio, PTH, 1,25-(OH)2 vitamin D3, FGF23, tubular threshold for phosphate reabsorption (TmP/GFR), plasma phosphate levels and the EQUIL2-based urinary supersaturation scores for calcium oxalate and brushite. For display clarity, only statistically significant differences ($p<0.05$) between groups are indicated, as determined by Kruskal-Wallis statistical test. *Abbreviations. B/LB, benign/likely benign; Br, brushite; CaOx, calcium oxalate; LP/P, likely pathogenic/pathogenic; TmP/GFR, ratio of tubular maximum reabsorption of phosphate (TmP) to glomerular filtration rate (GFR); VUS, variant of unknown significance.*

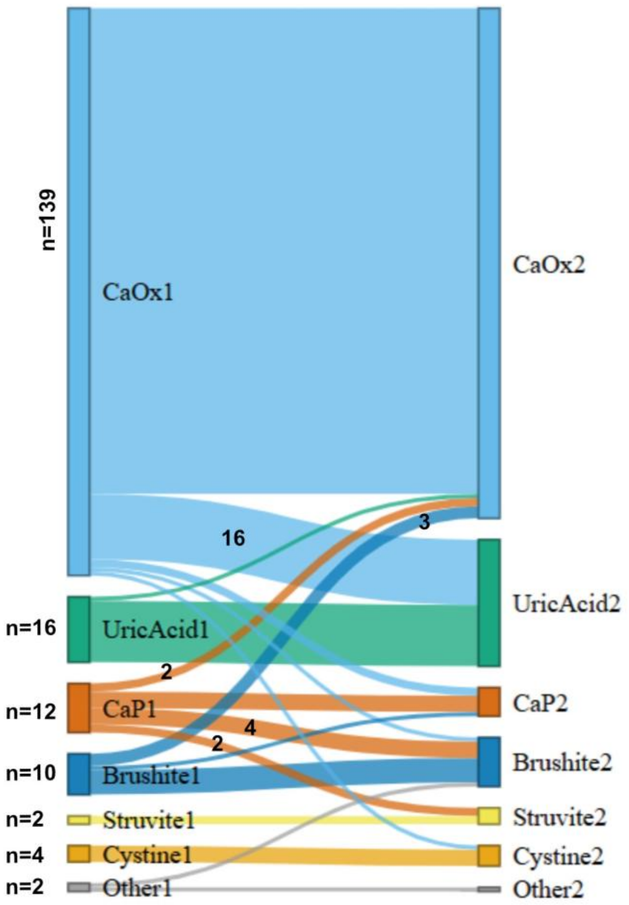

ID	gene	Stone episode 1	Stone episode 2
SKSC_01_0020	*SLC3A1 (b)*	Cystine	Cystine
SKSC_01_0022	*SLC34A3 (m)*	Brushite	Brushite
SKSC_01_0045	*SLC3A1 (b)*	Cystine	Cystine
SKSC_01_0060	*SLC34A3 (m)*	CaOx	Brushite
SKSC_01_0100	*SLC3A1 (b)*	Cystine	Cystine
SKSC_01_0133	*SLC7A9 (b)*	Cystine	Cystine
SKSC_01_0200	*SLC7A9 (m)*	CaOx	CaOx
SKSC_02_0012	*SLC4A1 (m)*	CaOx	CaOx
SKSC_02_0074	*SLC34A3 (m)*	Brushite	CaOx
SKSC_04_0070	*SLC4A1 (m)*	Brushite	Brushite
SKSC_06_0003	*MEN1 (m)*	Other	Brushite
SKSC_06_0013	*SLC2A9 (m)*	CaOx	CaOx
SKSC_05_0024	*SLC3A1 (b)*	CaOx	Cystine

Supplemental Figure 7: Kidney stone composition in recurrent stone episodes

The plot shows kidney stone composition at first (available) stone event on the left side of the graph (1) and on the second (available) stone event (2) on the right side of the graph. The number of KSF participants with each type of stone at timepoint 1 is indicated with n. The numbers on the crossing lines indicate the number of individuals where the stone type changed in the second episode. The table indicates the KSF with monogenic disease with the reported composition of the stones at each episode (m monoallelic, b biallelic).

Supplemental Table 1: Virtual panel for kidney stone associated genes, including OMIM (*) number (Online Mendelian Inheritance in Man https://omim.org/) and transcript number analyzed here (NM).

GENE SYMBOL	GENE NAME	OMIM (*)	NM
ADCY10	adenylate cyclase 10	605205	NM_001297772.2
AGXT	alanine-glyoxylate aminotransferase	604285	NM_000030.3
ALPL	alkaline phosphatase, liver	171760	NM_001369804.2
APRT	adenine phosphoribosyltransferase	102600	NM_000485.3
ATP6V0A4	ATPase, H+ transporting, lysosomal V0, subunit a4	605239	NM_130841.3
ATP6V1B1	ATPase, H+ transporting, lysosomal 56/58 kDa, V1, subunit B1	192132	NM_001692.4
ATP6V1C2	ATPase, H+ transporting, lysosomal, V1 subunit C2	618070	NM_001039362.2
BSND	barttin CLCNK-type accessory subunit beta	606412	NM_057176.3
CA2	carboanhydrase II	611492	NM_001293675.2
CASR	calcium sensing receptor	601199	NM_000388.4
CLCN5	H+/Cl- exchanger	300008	NM_001272102.2
CLCNKB	chloride channel, voltage sensitive Kb	602023	NM_000085.5
CLDN2	claudin 2	301060	NM_001171092.1
CLDN16	claudin 16	603959	NM_001378493.1
CLDN19	claudin 19	610036	NM_001123395.2
CYP24A1	cytochrome P450, family 24, subfamily A, polypeptide 1	126065	NM_000782.5
FAM20A	pseudokinase FAM20A	611062	NM_001243746.2
FOXI1	forkhead box protein I1	601093	NM_012188.5
GRHPR	glyoxylate reductase/hydroxypyruvate reductase	604296	NM_012203.2
HNF4A	hepatocyte nuclear factor 4α	600281	NM_001030004.3
HOGA1	4-hydroxy-2-oxoglutarate aldolase 1	613597	NM_001134670.2
HPRT1	hypoxanthine phosphoribosyltransferase 1	308000	NM_000194.3
KCNJ1	ATP-sensitive inward rectifier potassium channel 1	600359	NM_000220.6
MAGED2	Melanoma antigen, family D, 2	300470	NM_177433.3
MEN1	Menin 1	613733	NM_130802.3
OCRL	inositol polyphosphate-5-phosphatase OCRL-1	300535	NM_001587.4
SLC12A1	solute carrier family 12, member 1	600839	NM_001184832.2
SLC22A12	solute carrier family 22, member 12	607096	NM_153378.3
SLC26A1	solute carrier family 26, member 1	610130	NM_134425.4
SLC2A9	solute carrier family 2, member 9	606142	NM_020041.3
SLC34A1	solute carrier family 34, member 1	182309	NM_001167579.2
SLC34A3	solute carrier family 34, member 3	609826	NM_001177317.2
SLC3A1	solute carrier family 3, member 1	104614	NM_000341.4
SLC4A1	solute carrier family 4, anion exchanger, member 1	109270	NM_000342.4
SLC7A9	solute carrier family 7, member 9	604144	NM_001243036.2
SLC9A3R1	solute carrier family 9, member 3, regulating factor 1	604990	NM_004252.5
VDR	vitamin D receptor	601769	NM_001374661.1
WDR72	WD repeat containing protein 72	613214	NM_001277176.2
XDH	xanthine dehydrogense	607633	NM_000379.4

Supplemental Table 2: Clinical and biochemical details for Kidney stone formers harboring (likely) pathogenic variants in a KSD gene

Cystinuria

SKSC_ID	Gene	Zygosity	c.position	p.position	ACMG criteria°	ACMG§	ClinVar*	CADD	gnomAD MAF (all)	Age 1st KS	Stone composition	EQUIL2		
												CaOx	Br	UA
SKSC_01_0020	***SLC3A1***	hom.	c.1094G>T	p.(Arg365Leu)	PM2, PM5, PP2, PP3, PP5	LP	Pathogenic	32.0	8e-06	18.7	Cystine	# 1.5	# 0.6	# 0.04
SKSC_01_0045	***SLC3A1***	hom.	c.1711T>G	p.(Cys571Gly)	PM1, PM2, PP2, PP3	LP	.	37.0	4.1e-05	28.6	Cystine	0.5	0.1	0.1
	SLC9A3R1	het.	c.458G>A	p.(Arg153Gln)	PS3, PM2, PP5, PP3, BS2	LP	Pathogenic, VUS	23.5	0.00186					
	HOGA1	het.	c.208C>T	p.(Arg70*)	PVS1, PM2, PP5	P	Pathogenic	35.0	0.0019					
SKSC_01_0156	***SLC3A1***	hom.	c.1400T>C	p.(Met467Thr)	PS1, PM2, PM5, PM1, PM2, PP3, PP5, PP4	P	Pathogenic, likely pathogenic	25.7	0.0025	24.3	Cystine	1.6	2.3	0.3
SKSC_05_0022	***SLC3A1***	het.	c.1400T>C	p.(Met467Thr)	PS1, PM2, PM5, PM1, PM2, PP3, PP5	P	Pathogenic, likely pathogenic	25.7	0.0025	27.0	Cystine	# 3.6	# 2.6	# 0.1
		het.	c.1011G>A	p.(Pro337Pro)	PM2, PP3_strong, PP5	P	-	-	2e-05					
SKSC_05_0024	***SLC3A1***	compound het.	c.1424C>T	p.(Thr475Ile)	PM1, PM2_supporting, PP2, PP3, PP4	LP	.-	32.0	-	40.9	CaOx, CaP / CaOx, Cystine	8.1	0.0	1.3
			c.1400T>C	p.(Met467Thr)	PS1, PM2, PM5, PM1, PM2, PP3, PP5, PP4	P	Pathogenic, likely pathogenic	25.7	0.0025					
SKSC_01_0038	***SLC3A1***	het.	c.1400T>C	p.(Met467Thr)	PS1, PM2, PM5, PM1, PM2, PP3, PP5	P	Pathogenic, likely pathogenic	25.7	0.0025	31.6	CaOx	6.5	2.9	1.0
SKSC_01_0139	***SLC3A1***	het.	c.1400T>C	p.(Met467Thr)	PS1, PM2, PM5, PM1, PM2, PP3, PP5	P	Pathogenic, likely pathogenic	25.7	0.0025	32.6	CaOx	6.6	0.9	0.9
SKSC_01_0155	***SLC3A1***	het.	c.1400T>C	p.(Met467Thr)	PS1, PM2, PM5, PM1, PM2, PP3, PP5	P	Pathogenic, likely pathogenic	25.7	0.0025	10.3	CaOx, CaP	3.8	3.7	0.3
SKSC_01_0165	***SLC3A1***	het.	c.1400T>C	p.(Met467Thr)	PS1, PM2, PM5, PM1, PM2, PP3, PP5	P	Pathogenic, likely pathogenic	25.7	0.0025	40.9	CaOx	5.4	0.7	1.6
SKSC_01_0167	***SLC3A1***	het.	c.808C>T	p.(Arg270*)	PVS1, PM2, PP5	LP	Pathogenic	37.0	0.0004	30.4	CaOx	8.4	1.7	1.2

SKSC_01_0193	***SLC3A1***	het.	c.1366C>T	p.(Arg456Cys)	PM2, PP3, PP2, PP5, PP4	LP	-	34.0	2.4e-05	30.1	Cystine	1.5	1.4	0.1
SKSC_02_0150	***SLC3A1***	het.	c.1322C>T	p.(Pro441Leu)	PM2, PP2, PP3_strong	LP	-	34.0	4e-06	62.0	NA	4.4	0.2	6.0
SKSC_02_0212	***SLC3A1***	het.	c.1136+2T>C	?	PVS1, PM2	LP	-	10.42	8.5e-05	55.4	NA	16.6	1.2	3.6
SKSC_02_0215	***SLC3A1***	het.	c.1400T>C	p.(Met467Thr)	PS1, PM2, PM5, PM1, PM2, PP3, PP5	P	Pathogenic, likely pathogenic	25.7	0.0025	42.0	NA	13.7	3.4	1.6
SKSC_02_0221	***SLC3A1***	het.	c.1400T>C	p.(Met467Thr)	PS1, PM2, PM5, PM1, PM2, PP3, PP5	P	Pathogenic, likely pathogenic	25.7	0.0025	75.9	CaOx	5.9	0.1	2.1
SKSC_02_0264	***SLC3A1***	het.	c.1400T>C	p.(Met467Thr)	PS1, PM2, PM5, PM1, PM2, PP3, PP5	P	Pathogenic, likely pathogenic	25.7	0.0025	39.4	CaOx	15.2	0.9	3.0
SKSC_04_0128	***SLC3A1***	het.	c.647C>T	p.(Thr216Met)	PM2, PP2, PP3_strong, PP5	P	Pathogenic	33.0	8.9e-05	54.3	NA	2.8	0.2	0.6
SKSC_05_0010	***SLC3A1***	het.	c.1322C>T	p.(Pro441Leu)	PM2, PP2, PP3_strong	LP	-	34.0	4e-06	38.3	CaOx	7.1	13.0	0.7
SKSC_06_0006	***SLC3A1***	het.	c.1400T>C	p.(Met467Thr)	PS1, PM2, PM5, PM1, PM2, PP3, PP5	P	Pathogenic, likely pathogenic	25.7	0.0025	39.7	CaOx, CaP	4.0	1.1	0.3
SKSC_01_0100	***SLC7A9***	het.	c.502C>G	p.(Leu168Val)	PM1, PM2, PP2, PP4	LP	-	10.98	8e-06	49.8	Cystine	2.5	2.2	0.1
		het.	c.505_508delAGCG	p.(Ser169Cysfs*17)	PVS1, PM2, PP4	LP	-	.	4e-06					
		het.	c.1293C>A	p.(Ser431Arg)	PM2, PP2, PP4	VUS	-	12.01						
SKSC_05_0066	***SLC7A9***	het.	c.1060G>A	p.(Ala354Thr)	PM2, PP3_strong, PP2, PP5	LP	-	33.0	1.2e-05	31.1	CaOx	7.5	4.1	1.9
		het.	c.544G>A	p.(Ala182Thr)	PM1, PM2, PP2, PP5_verystrong	P	Pathogenic	18.3	0.0026					
SKSC_06_0010	***SLC7A9***	compound het.	c.544G>A	p.(Ala182Thr)	PM1, PM2, PP2, PP5_verystrong	LP	Pathogenic	18.3	0.0026	2.1	Cystine	4.7	2.5	0.1
			c.505_508delAGCG	p.(Ser169Cysfs*17)	PVS1, PM2	LP	-	-	4e-06					
			c.502C>G	p.(Leu168Val) *in cis with p.(S169Cfs*17)*	PM1, PM2, PP2	VUS	-	10.98	8e-06					
	BSND	het.	c.139G>A	p.(Gly47Arg)	PM2, PP5_verystrong	LP	Pathogenic	28.3	9.8e-05					
SKSC_01_0133	***SLC7A9***	compound het.	c.614dupA	p.(Asn206Glufs*3)	PVS1, PM2, PP5, PP4	P	Pathogenic	-	0.0001	26.0	CaOx / Cystine	7.4	4.2	0.2
			c.605-3C>A	?	PS3, PM2, PP3, PP4	LP	VUS	17.8	4e-06					

SKSC_01_0106	***SLC7A9***	het.	c.544G>A	p.(Ala182Thr)	PM1, PM2, PP2, PP5	P	Pathogenic	18.3	0.0026	24.9	CaOx	3.0	1.5	0.3
SKSC_01_0200	***SLC7A9***	het.	c.209C>T	p.(Ala70Val)	PM1, PM2, PP2, PP5	LP	Likely pathogenic	16.43	2e-05	39.6	CaOx, CaP	11.4	4.6	0.2
SKSC_02_0120	***SLC7A9***	het.	c.544G>A	p.(Ala182Thr)	PM1, PM2, PP2, PP5	P	Pathogenic	18.3	0.0026	35.1	CaOx	1.9	0.7	0.1
SKSC_03_0037	***SLC7A9***	het.	c.313G>A	p.(Gly105Arg)	PM1, PM2, PM5, PP2, PP3, PP5	P	Pathogenic, likely pathogenic	33.0	0.0003	38.5	NA	7.5	0.0	1.4
SKSC_04_0062	***SLC7A9***	het.	c.544G>A	p.(Ala182Thr)	PM1, PM2, PP2, PP5	LP	Pathogenic	18.3	0.0026	33.6	CaOx, CaP	5.9	3.4	0.5
SKSC_04_0072	***SLC7A9***	het.	c.313G>A	p.(Gly105Arg)	PM1, PM2, PM5, PP2, PP3, PP5	LP	Pathogenic, likely pathogenic	33.0	0.0003	54.2	NA	4.8	6.9	0.3
SKSC_04_0130	***SLC7A9***	het.	c.544G>A	p.(Ala182Thr)	PM1, PM2, PP2, PP5	P	Pathogenic	18.3	0.0026	22.4	CaOx, CaP	2.7	4.0	0.4
SKSC_05_0102	***SLC7A9***	het.	c.544G>A	p.(Ala182Thr)	PM1, PM2, PP2, PP5	LP	Pathogenic	18.3	0.0026	52.8	CaOx, CaP	3.2	0.2	0.0
	BSND	het.	c.139G>A	p.(Gly47Arg)	PS1, PM2	LP	Pathogenic	28.3	9.8e-05					
SKSC_05_0012	***SLC7A9***	het.	c.1283C>T	p.(Pro428Leu)	PM2, PP3_moderate, PP2, PP4	LP	-	26.4	-	66.6	Other	# 6.3	# 0.0	# 2.7

Renal phosphate wasting

**SKSC_ID**	**Gene**	**Zygosity**	**c.position**	**p.position**	**ACMG criteria**	**ACMG**	**ClinVar**	**CADD**	**gnomAD**	**Age 1st KS**	**Stone**	**EQUIL2**		
												**CaOx**	**Br**	**UA**
SKSC_01_0103	***SLC34A1***	het.	c.891delG	p.(Asn298Thrfs*106)	PVS1, PM2, PP4	P	-	-	-	44.2	CaOx, CaP	5.5	1.5	0.3
SKSC_02_0084	***SLC34A1***	het.	c.460_480dupATCCTGGTGACCGTGCTGGTG	p.(Ile154_Val160dup)	PM2, PM4, PM1, PP5	LP	Pathogenic	-	2e-05	42.6	CaOx,Uric Acid	4.8	2.2	4.4
SKSC_02_0124	***SLC34A1***	het.	c.643C>T	p.(Arg215Trp)	PM2, PP3, PP5, PP4	LP	-	33.0	8.2e-05	38.8	NA	# 4.7	# 0.2	# 0.7
SKSC_01_0022	***SLC34A3***	het.	c.1058G>T	p.(Arg353Leu)	PM2, PP5_strong	LP	Pathogenic	23.4	1.7e-05	22.6	CaOx, CaP			
SKSC_01_0060	***SLC34A3***	het.	c.304+2T>C		PVS1, PM2, PM3, PP5	P	Pathogenic	23.6	3.7e-05	37.5	CaOx, CaP	4.8	3.5	0.5
SKSC_01_0195	***SLC34A3***	het.	c.304+2T>C		PVS1, PM2, PP5	P	-	23.6	3.7e-05	24.5	CaOx, CaP	7.0	4.5	0.3
SKSC_02_0023	***SLC34A3***	het.	c.133delT	p.(Trp45Glyfs*5)	PVS1, PM2	LP	-	-	-	25.0	CaOx	9.3	2.1	0.9
SKSC_02_0022[a]	***SLC34A3***	het.	c.560+23_	intronic	PM2, PP5_strong	LP	VUS	-	0.0002	34	NA	# 3.5	# 0.03	# 3.3

			561-42delGGGCTGGGGCTGCAGTGGCAGCCCCAGCCC											
SKSC_02_0061	***SLC34A3***	het.	c.1248_1249delCT	p.(Leu417Thrfs*175)	PVS1, PM2, PP5	P	-	-	6.1e-05	18.9	CaOx, CaP	4.9	5.4	0.7
SKSC_02_0074	***SLC34A3***	het.	c.894_925+69del	p.(Asn299Profs*282)	PVS1, PP5, PP4	P	-	-	1.3e-05	11.8	CaP	4.2	4.7	0.0
SKSC_05_0009	***SLC34A3***	het.	c.560+23_561-42delGGGCTGGGGCTGCAGTGGCAGCCCCAGCCC	intronic	PM2, PP5_strong	LP	VUS	-	0.0002	20	NA	4.0	1.8	2.7
SKSC_05_0086	***SLC34A3***	het.	c.304+2T>C	?	PVS1, PM2, PP5	P	-	23.6	3.7e-05	61.3	CaOx, Other	9.8	0.8	1.6
SKSC_01_0151	***SLC9A3R1***	het.	c.458G>A	p.(Arg153Gln)	PS3, PM2, PP5, PP3	LP	Pathogenic, VUS	35.0	0.0019	22.5	Uric Acid	4.4	0.1	2.3
SKSC_02_0004[a,b]	***SLC9A3R1***	het.	c.458G>A	p.(Arg153Gln)	PS3, PM2, PP5, PP3	LP	Pathogenic, VUS	35.0	0.0019	28.3	CaP	NA	NA	NA
SKSC_05_0119	***SLC9A3R1***	het.	c.458G>A	p.(Arg153Gln)	PS3, PM2, PP5, PP3	LP	Pathogenic, VUS	35.0	0.0019	29.7	Uric Acid, Other	6.8	0.4	3.1
SKSC_05_0133	***SLC9A3R1***	het.	c.888+2T>C	?	PVS1, PM2	LP	-	23.2	0.0002	20.4	CaOx	5.8	0.5	1.8
SKSC_03_0009	***SLC9A3R1***	het.	c.458G>A	p.(Arg153Gln)	PS3, PM2, PP5, PP3	LP	Pathogenic, VUS	35.0	0.0019	60.8	CaOx	# 6.6	# 1.4	# 5.1

Calcium metabolism

SKSC_ID	Gene	Zygosity	c.position	p.position	ACMG criteria	ACMG	ClinVar	CADD	gnomAD	Age 1st KS	Stone	EQUIL2		
												CaOx	Br	UA
SKSC_04_0075	***ADCY10***	het.	c.579C>A	p.(Cys193*)	PVS1, PM2	LP	-	38.0	4e-06	58.7	CaP, Other	2.6	2.1	0.2
	SLC3A1	het.	c.769A>G	p.(Ser257Gly)	PP3_strong, PM2, PP2	LP	-	26.6	2e-05					
SKSC_02_0243	***ALPL***	het.	c.571G>A	p.(Glu191Lys)	PM1, PM2, PM5, PP2, PP3, PP5	LP	Pathogenic, likely pathogenic	29.2	0.0026	38.5	CaOx, CaP	9.6	6.9	0.2
SKSC_04_0054	***ALPL***	het.	c.984_986delCTT	p.(Phe328del)	PM1, PM2, PM4, PP5	LP	-	-	1.6e-05	47.9	CaOx, CaP	4.8	5.5	0.5
SKSC_02_0042[a]	***ALPL***	het.	c.571G>A	p.(Glu191Lys)	PM1, PM2, PM5, PP2, PP3, PP5	LP	Pathogenic|other	29.2	0.0026	30.5	CaOx	5.0	0.9	0.2
SKSC_01_0071	***ALPL***	het.	c.918C>G	p.(Asp306Glu)	PM1, PM2, PM5, PP2, PP3	LP	-	23.2	-	22.1	CaOx, CaP	3.5	1.9	0.4

SKSC_02_0094[a]	***ALPL***	het.	c.1471G>A	p.(Gly491Arg)	PM1, PM2, PP5, PP2, PP3	LP	-	27.7	8e-06	73.7	CaOx, CaP	3.7	0.6	0.8
SKSC_05_0028	***ALPL***	het.	c.1574delG	p.Phe524_Ter 525insTer	PM2, PVS1	LP	-	33	0.0003	NA	NA	5.5	0	0.5
SKSC_01_0125	***ALPL***	het.	c.1574delG	p.Phe524_Ter 525insTer	PM2, PVS1	LP	-	33	0.0003	41.3	Uric Acid	3.4	0.1	1.7
SKSC_05_0138	***ALPL***	het.	c.1574delG	p.Phe524_Ter 525insTer	PM2, PVS1	LP	-	33	0.0003	55.0	CaOx, CaP	4.0	0.6	1.2
SKSC_01_0016	***ATP6V1B1***	het.	c.1037C>G	p.(Pro346Arg)	PP3_strong, PM2, PP5	P	Pathogenic	28.8	1.6e-05	59.4	Uric Acid	2.3	0.9	2.1
SKSC_05_0007	***ATP6V0A4***	het.	c.2458G>A	p.(Gly820Arg)	PM1, PM2, PP3_moderate	LP	Pathogenic	33.0	4.9e-05	23.0	CaOx	2.5	0.0	3.1
SKSC_05_0029	***ATP6V0A4***	het.	c.2011-1G>A	?	PVS1, PM2	LP	-	14.69	-	50.7	CaOx, CaP	# 2.6	# 1.0	# 2.7
SKSC_02_0026[a]	***BSND***	het.	c.685C>T	p.(Gln229*)	PVS1, PM2	LP	-	23.2	8e-06	30.1	CaOx	NA	NA	NA
SKSC_01_0145	***CASR***	het.	c.269A>C	p.(Asn90Thr)	PM1, PM2, PP2, PP3	LP	Likely pathogenic	24.6	8e-06	23.4	CaOx	9.8	1.2	2.2
SKSC_06_0011	***CASR***	het.	c.275C>T	p.(Thr92Met)	PM1, PP2, PM2, PP3	LP	-	26.1	4e-06	35.0	NA	9.7	3.4	0.1
SKSC_05_0082	***CASR***	het.	c.308C>T	p.(Thr103Ile)	PM1, PM2, PP2, PP3	LP	-	25.0	8e-06	22.6	CaOx, CaP	2.7	0.7	0.2
SKSC_02_0093	***CYP24A1***	hom.	c.428_430d elAAG	p.(Glu143del)	PM2, PM3, PM4, PP5	P	Pathogenic, VUS	-	0.0005	24.4	CaOx, CaP	4.9	7.0	0.2
SKSC_02_0173	***CYP24A1***	hom.	c.428_430d elAAG	p.(Glu143del)	PM2, PM3, PM4, PP5	P	Pathogenic, VUS	-	0.0005	26.5	CaOx, CaP	6.7	2.3	0.3
SKSC_01_0053	***CYP24A1***	het.	c.667A>T	p.(Arg223*)	PVS1, PM2	LP	-	37.0	1.6e-05	46.8	CaOx, CaP	2.2	0.5	1.3
SKSC_01_0083	***CYP24A1***	het.	c.428_430d elAAG	p.(Glu143del)	PM2, PM3, PM4, PP5	P	Pathogenic, VUS	-	0.0005	23.6	CaOx, CaP	2.1	0.1	0.6
SKSC_01_0094	***CYP24A1***	het.	c.1186C>T	p.(Arg396Trp)	PM2, PM5, PP3, PP5	P	Pathogenic	33.0	0.0007	22.0	CaOx, CaP	3.6	5.4	0.4
SKSC_01_0102	***CYP24A1***	het.	c.1187G>A	p.(Arg396Gln)	PM2, PM5, PP2, PP5, PP3	LP	-	35.0	7.3e-05	59.0	CaP	6.7	4.5	1.3
SKSC_01_0140	***CYP24A1***	het.	c.1540C>T	p.(Arg514*)	PM2, PVS1	LP	-	37.0	3.3e-05	19.1	CaOx	14.3	3.0	0.5
SKSC_02_0099[a]	***CYP24A1***	het.	c.1187G>A	p.(Arg396Gln)	PM2, PM5, PP2, PP5, PP3	LP	-	35.0	7.3e-05	35.9	CaOx	4.0	0.2	3.4
SKSC_02_0154	***CYP24A1***	het.	c.1186C>T	p.(Arg396Trp)	PM2, PM5, PP3, PP5_very strong	P	Pathogenic	33.0	0.0007	31.4	CaOx, CaP	5.6	1.5	1.2
SKSC_05_0097	***CYP24A1***	het.	c.476G>A	p.(Arg159Gln)	PM2, PP3, PP5	LP	Pathogenic	34.0	2.8e-05	NA	NA	2.8	0.4	1.5
SKSC_02_0180	***CLCNKB***	het.	c.49C>T	p.(Gln17*)	PVS1, PM2	LP	-	8.288	-	33.7	CaOx, CaP	8.3	0.6	2.7
SKSC_03_0004	***CLCNKB***	het.	c.15_19dup CCTCC	p.(Leu7Profs*21)	PVS1, PM2, BS2	LP	-	-	0.0003	59.6	CaOx	# 8.4	# 3.3	# 5.2
SKSC_03_0019	***CLCNKB***	het.	c.235C>T	p.(Gln79*)	PVS1, PM2	LP	-	26.5	-	61.4	NA	7.3	0.7	7.0
SKSC_05_0006	***CLCNKB***	het.	c.97_100du pGTCA	p.(Thr34Serfs*80)	PVS1, PM2	LP	-	-	8e-06	NA	NA	3.6	0.0	6.0
SKSC_05_0040	***CLCNKB***	het.	c.498+1G>C	?	PVS1, PM2	LP		23.0	4e-06	31.1	CaOx	4.2	0.0	0.9

SKSC_01_0079	***CLDN16***	het.	c.217+5G>A	?	PM2, PP3, PP5_strong	LP	-	-	1.6e-05	23.2	CaOx	2.3	0.9	0.2
SKSC_01_0054	***KCNJ1***	het.	c.590T>A	p.(Leu197His)	PP3_strong, PM2, PP2	LP	-	26.4	-	27.5	CaOx, CaP	4.5	2.0	0.8
SKSC_04_0030	***MEN1***	het.	c.694C>T	p.(Arg232Cys)	PM1, PM2, PP2, PP3	LP	-	34.0	4e-06	23.2	NA	NA	NA	NA
SKSC_06_0003	***MEN1***	het.	c.982C>A	p.(His328Asn)	PM1, PM2, PP2, PP3	LP	-	22.4	-	43.2	CaOx, CaP, Other	2.6	1.0	0.1
SKSC_04_0135	***OCRL***	hemi.	c.863A>G	p.(Tyr288Cys)	PM2, PP3_moderate, PP2, PP4	LP	-	26.8	-	45.3	CaOx	8.3	0.8	2.8
SKSC_02_0265	***SLC4A1***	het.	c.713A>T	p.(Glu238Val)	PM2, PP5_strong	LP	-	22.6	0.0016	19.2	CaOx	10.5	1.3	1.8
SKSC_04_0070	***SLC4A1***	het.	c.2726T>C	p.(Met909Thr)	PS3, PM2, PP3, PP5	LP	likely pathogenic	24.2	-	16.8	CaOx	9.6	7.7	0.3
SKSC_02_0012	***SLC4A1***	het.	c.1166G>A	p.(Arg389His)	PP3_strong, PM2, PP4	LP	-	35.0	1.2e-05	61.7	CaOx	# 3.4	# 0.8	# 0.01
SKSC_02_0127[a, b]	***SLC4A1***	het.	c.1166G>A	p.(Arg389His)	PP3_strong, PM2, PP4	LP	-	35.0	1.2e-05	34.7	CaOx, CaP	5.2	3.3	0.0
	FAM20A	het.	c.727C>T	p.(Arg243*)	PVS1, PM2	P	-	41.0	2.4e-05					
SKSC_02_0097[a]	***SLC12A1***	het.	c.2712_2713delGA	p.(Lys905Thrfs*6)	PVS1, PM2	LP	-	-	-	26.9	CaOx, CaP	4.7	2.4	0.9
SKSC_04_0145	***SLC12A1***	het.	c.704C>T	p.(Thr235Met)	PP3_strong, PM2	LP	-	34.0	0.0001	39.9	CaOx	4.5	1.0	0.8
SKSC_04_0047	***VDR***	het.	c.218G>A	p.(Arg73Gln)	PM2, PP3_moderate, PP5	LP	Pathogenic	35.0	3.7e-05	34.3	CaOx, CaP,Other	4.2	0.6	3.7

Uric Acid

SKSC_ID	Gene	Zygosity	c.position	p.position	ACMG criteria	ACMG	ClinVar	CADD	gnomAD	Age 1st KS	Stone	EQUIL2		
												CaOx	Br	UA
SKSC_01_0081	***SLC22A12***	het.	c.1070G>A	p.(Trp357*)	PVS1, PM2, PP5	P	Pathogenic	35.0	1.7e-05	30.7	CaOx	7.9	0.6	2.7
SKSC_02_0247	***SLC2A9***	het.	c.1343C>T	p.(Pro448Leu)	PM2, PP3, PP5_strong	LP	-	27.4	6.5e-05	22.9	NA	5.5	0.1	1.6
SKSC_03_0015	***SLC2A9***	het.	c.1409delC	p.(Pro470Hisfs*13)	PVS1, PM2	LP	-	-	-	NA	NA	9.0	4.5	1.0
SKSC_06_0013	***SLC2A9***	het.	c.682-2A>G	?	PM2, PVS1	LP	-	22.9	-	52.1	CaOx	0.8	0.0	2.8

Hyperoxaluria

SKSC_ID	Gene	Zygosity	c.position	p.position	ACMG criteria	ACMG	ClinVar	CADD	gnomAD	Age 1st KS	Stone	EQUIL2		
												CaOx	Br	UA
SKSC_02_0147	***AGXT***	het.	c.82C>T	p.(Pro28Ser)	PM2, PM1, PP2, PP3	LP	-	24.7	3.3e-05	36.8	NA	11.7	1.1	8.5

SKSC_03_0018	***AGXT***	het.	c.508G>A	p.(Gly170Arg)	PM1, PM2, PP2, PP3, PP5	P	Pathogenic/ Likely pathogenic	27.0	0.0005	45.0	NA	NA	NA	NA
SKSC_04_0114	***AGXT***	het.	c.247C>T	p.(His83Tyr)	PM1, PM2, PM5, PP2, PP3	LP	-	23.8	**-**	34.4	CaOx, CaP	3.3	3.1	1.5

Other

**SKSC_ID**	**Gene**	**Zygosity**	**c.position**	**p.position**	**ACMG criteria**	**ACMG**	**ClinVar**	**CADD**	**gnomAD**	**Age 1st KS**	**Stone**	**EQUIL2**		
												**CaOx**	**Br**	**UA**
SKSC_01_0092	***SLC26A1***	het.	c.1225G>A	p.(Gly409Ser)	PM2, PP3_strong	LP	-	28.1	2e-05	52.1	CaOx, CaP	5.5	2.0	0.2
SKSC_01_0144	***SLC26A1***	het.	c.298A>G	p.(Arg100Gly)	PM2, PM5, PP2, PP3	LP	**-**	18.32	5.6e-05	28.0	CaOx	12.4	2.4	2.1
SKSC_04_0051	***SLC26A1***	het.	c.1465G>A	p.(Gly489Arg)	PP3_strong, PM2	LP	-	26.3	6.1e-05	44.9	CaOx,Uric Acid	# 4.8	# 1.8	# 1.5

° indicates the American College of Medical Genetics and Genomics (ACMG) criteria fulfilled to reach the classification shown in column [§]. * indicates the classification of the variant in ClinVar (https://www.ncbi.nlm.nih.gov/clinvar/). The minor allele frequency (MAF) in GnomAD v2.1.1 (https://gnomad.broadinstitute.org/) is indicated for the aggregated ethnicities (all). [a] indicates subjects also enrolled in the Bern Kidney Stone Registry; [b] indicates individuals/variants reported in (Anderegg et al.). Multiple variants present in the same individual are indicated in consecutive lines. Variants considered as causative in a "monogenic model" are highlighted in light green. Variants initially considered as causative in heterozygosity but reclassified as insufficient to cause monogenic KSD after this work based on lack of biochemical consequences are highlighted in light pink. Monoallelic ("het") variants in recessive genes are not highlighted (white rows). The stone composition is indicated for the first stone available. EQUIL2 scores were calculated at baseline (V2) except when marked with #. ***Abbreviations***: CADD, Combined Annotation Dependent Depletion; hemi, hemizygous; het., heterozygous; hom., homozygous; LP, likely pathogenic; n, number; P, pathogenic; VUS, variant of uncertain significance.

Supplemental Table 3: (Likely) pathogenic variants identified in non-Kidney Stone formers (NKSF)

Condition	SKSC_ID	Gene	Zygosity	c.position	p.position	ACMG criteria°	ACMG§	ClinVar#	CADD	gnomAD
Cystinuria	SKSC_09_0056	***SLC3A1***	het.	c.1400T>C	p.Met467Thr	PS1, PM2, PM5, PM1, PM2, PP3, PP5	P	Pathogenic, likely pathogenic	25.7	0.0025
	SKSC_09_0117	***SLC7A9***	het.	c.1147T>C	p.Trp383Arg	PM2, PP3_moderate, PP2, PP4	LP	.	25.1	-
	SKSC_09_0095	***SLC7A9***	het.	c.943G>A	p.Gly315Ser	PP3_strong, PM2, PP2	LP	.	34.0	1.6e-05
	SKSC_09_0120	***SLC7A9***	het.	c.544G>A	p.Ala182Thr	PM1, PM2, PP2, PP5	P	Pathogenic	18.3	0.0026
	SKSC_09_0125	***SLC7A9***	het.	c.313G>A	p.Gly105Arg	PM1, PM2, PM5, PP2, PP3, PP5	P	Pathogenic	33.0	0.0003
		SLC9A3R1	het.	c.458G>A	p.Arg153Gln	PS3, PM2, PP5, PP3	LP	Pathogenic, VUS	35.0	0.0019
	SKSC_09_0144	***SLC7A9***	het.	c.217G>A	p.Gly73Arg	PS1, PM2, PM1_supporting, PP2, PP3, PP4	LP	.	28.9	0.0
Renal phosphate wasting	SKSC_09_0052	***SLC9A3R1***	het.	c.458G>A	p.Arg153Gln	PS3, PM2, PP5, PP3	LP	Pathogenic, VUS	35.0	0.0019
	SKSC_09_0188	***SLC9A3R1***	het.	c.458G>A	p.Arg153Gln	PS3, PM2, PP5, PP3	LP	Pathogenic, VUS	35.0	0.0019
		SLC3A1	het.	c.1400T>C	p.Met467Thr	PS1, PM2, PM5, PM1, PM2, PP3, PP5	P	Pathogenic, likely pathogenic	25.7	0.0025
	SKSC_09_0074	***SLC34A1***	het.	c.891delG	p.Asn298fs	PVS1, PM2, PP4	P	.	-	-
	SKSC_09_0003	***SLC34A1***	het	c.713A>C	p.Glu238Ala	PM2, PP3_moderate, PP5_strong	LP	Pathogenic	26.9	-
Calcium metabolism	SKSC_09_0175	***ALPL***	het.	c.119C>T	p.Ala40Val	PM2, PM1_supporting, PP2, PP3, PP5	LP	.	23.6	4e-06
	SKSC_09_0099	***CYP24A1***	het.	c.1206delA	p.Val403fs	PVS1, PM2	LP	.	-	-
	SKSC_09_0149	***CYP24A1***	het.	c.1226T>C	p.Leu409Ser	PM2, PP5	LP	Pathogenic	31.0	0.0008
	SKSC_09_0058	***KCNJ1***	het.	c.88T>C	p.Cys30Arg	PP3_strong, PM2	LP	.	25.2	8e-06
Other	SKSC_09_0078	***FAM20A***	het.	c.34_35delCT	p.Leu12fs	PVS1, PM2, PP5	P	Pathogenic	-	1.3e-05
	SKSC_09_0124	***HOGA1***	het.	c.700+5G>T	?	PM2, PP3, PP5	P	Pathogenic	-	0.0012
	SKSC_09_0103	***SLC26A1***	het.	c.1244C>T	p.Ser415Phe	PP3_strong, PM2	LP	.	25.7	0.0004
	SKSC_09_0009	***SLC26A1***	het.	c.400G>A	p.Gly134Arg	PM2, PP3_strong	LP	.	29.4	0.0001
	SKSC_09_0200	***XDH***	het.	c.2322+1delG	?	PVS1, PM2	LP	.	-	-

° indicates the American College of Medical Genetics and Genomics (ACMG) criteria fulfilled to reach the classification shown in column §. # indicates the classification of the variant in ClinVar (https://www.ncbi.nlm.nih.gov/clinvar/). The minor allele frequency (MAF) in GnomAD v2.1.1 (https://gnomad.broadinstitute.org/) is indicated for the aggregated ethnicities (all). Multiple variants present in the same individual are indicated in consecutive lines. Variants considered as causative in a "monogenic model" in heterozygosity are highlighted in light green. Variants initially considered to cause monogenic KSD in heterozygosity but reclassified as insufficient to cause monogenic KSD after this work based on lack of biochemical alterations are highlighted in light pink. Monoallelic ("het") variants in recessive genes are not highlighted (white rows). The stone composition is indicated for the first stone available. *Abbreviations: CADD, Combined Annotation Dependent Depletion; hemi, hemizygous; het., heterozygous; LP, likely pathogenic; n, number; P, pathogenic.*

Supplemental Table 4: **LP/P variants recurring more than twice in KSF and/or NKSF**

Gene	c.position / p.position	KSF<br>n= 701	NKSF<br>n= 200	MAF<br>GnomAD	p value<br>compared to GnomAD<br>(KSF / NKSF)
SLC9A3R1	c.458G>A / p.(Arg153Gln)	6 (0.0086)	3 (0.014913)	0.00186	<.0001/ <0.000019
SLC7A9	c.544G>A / p.(Ala182Thr)	7 (0.01)	1 (0.005)	0.00257	<0.00001 / <0.00001
SLC34A3	c.304+2T>C / ?	3 (0.0042)	-	0.000039	<0.0001 / -
SLC3A1	c.1400T>C / p.(Met467Thr)	10 (0.0142)	2 (0.00995)	0.00241	< 0.00001 / 0.029684

Variants recurring in more than two individuals are displayed with the number of KSF and NKSF (% in brackets) harboring the indicated variant. The minor allele frequency (MAF) for each variant in GnomAD v2.1.1 is also indicated, with comparison with the frequency in KSF respectively NKSF in this cohort. Chi-square contingency table test was used for statistical comparison. - indicates that the variant was not identified in NKSF. Note that the *SLC9A3R1* variant p.(Arg153Gln) is more significantly enriched in NKSF than in KSF in our cohort and the *SLC7A9* variant p.(Ala182Thr) is equally enriched in both local cohorts compared to GnomAD. In contrast, the *SLC34A3* c.304+2T>C variant is significantly enriched in KSF compared to GnomAD and not identified in NKSF here, supporting its clinical relevance. Likewise, the *SLC3A1* p.(Met467Thr) variant is strongly enriched in KSF compared to both NKSF and GnomAD, also supporting its clinical relevance.

Supplemental Table 5: Urine concentration of the amino acids arginine, cystine, lysine and ornithine for KSF and NKSF with (likely) pathogenic variants detected in *SLC3A1* and *SLC7A9* and a double-sized matched control group

	**Arginine mmol/mol**	**p**	**Cystine mmol/mol**	**p**	**Lysine mmol/mol**	**p**	**Ornithine mmol/mol**	**p**
**Matched controls (n=126)**	2.09 [1.26/3.09]		5.11 [3.66/6.99]		10.34 [6.06/19.36]		2.33 [1.23/4.11]	
***SLC3A1* biallelic**	220.19 [141.75/268.54]	**<0.000**1	112.64 [81.76/128.38]	**<0.0001**	483.05 [272.94/491.95]	**<0.0001**	143.53 [129.19/159.28]	**<0.0001**
***SLC3A1* - LP/P het.**	2.93 [2.01/12.88]	**0.0304**	5.21 [4.62/11.31]	0.2413	13.03 [10.45/32.61]	0.0946	3.25 [1.89/5.18]	0.1889
***SLC3A1* - VUS het.**	3.67 [2.47/4.25]	0.0536	4.22 [3.93/5.08]	0.3500	13.34 [9.12/20.43]	0.4840	1.52 [1.13/2.49]	0.3355
***SLC7A9* - biallelic**	131.09 [5.75/336.00]	**0.0046**	53.78 [18.14/72.46]	**0.0006**	211.82 [77.99/420.93]	**0.0016**	72.46 [6.26/115.04]	**0.0003**
***SLC7A9* - LP/P het**	11.05 [2.44/15.51]	0.1083	31.97 [29.25/43.97]	**0.0001**	113.35 [99.45/156.51]	**<0.0001**	18.60 [16.31/23.10]	**0.0148**
***SLC7A9* - p.A182T het**	2.07 [2.06/2.42]	0.2261	5.08 [3.57/6.42]	0.2063	33.46 [12.82/42.94]	0.3372	2.92 [1.34/4.47]	0.6630
***SLC7A9* - VUS het**	3.76 [2.49/4.90]	0.4810	9.86 [7.53/10.81]	0.7226	24.73 [7.76/48.78]	0.0777	2.63 [2.08/3.37]	0.7226

Matched controls include age- and sex-matched KSF without variants in *SLC3A1* and/or *SLC7A9*. Within the control group, 65.9% (n=83) were male and median age at first kidney stone episode was 40.1 years. None of the controls had a documented cystine-containing stone or variants in cystine-handling genes. The concentrations are given in mmol/mol urine creatinine and expressed as median [25th / 75th percentile]. For statistical analyses, the Kruskal-Wallis test was applied. *Abbreviations: het., heterozygous; KSF, kidney stone formers; n, number.*

Supplemental Table 6: Genetic variants and urinary amino acid levels in KSF with cystine stones

SKSC_ID	% cystine stone	n stone episodes documented	gene variant 1	gene variant 2	segregation confirmed	Arginine (umol/L)	Cystine (umol/L)	Lysine (umol/L)	Ornithine (umol/L)
SKSC_01_0020	100	2	***SLC3A1*** c.1094G>T, p.(Arg365Leu)	***SLC3A1*** c.1094G>T, p.(Arg365Leu)	yes (hmz)	5163.04	2273.66	6376.27	1705.28
SKSC_01_0045	100	3	***SLC3A1*** c.1711T>G, p.(Cys571Gly)	***SLC3A1*** c.1711T>G, p.(Cys571Gly)	yes (hmz)	1691.8	515.1	1719.5	904.25
SKSC_01_0100	100	2	***SLC7A9*** p.Leu168Val; p.Ser169fs	***SLC7A9*** p.Ser431Arg	no	3158.38	505.56	1991.08	2337.86
SKSC_01_0133	100	2	***SLC7A9*** c.605-3C>A, p.(?)	***SLC7A9*** c.614dupA, p.(p.Asn206fs)	yes	2951.17	553.26	5515.88	553.26
SKSC_01_0156	100	1	***SLC7A9*** c.1400T>C, p.(Met467Thr)	***SLC7A9*** c.1400T>C, p.(p.Met467Thr)	yes (hmz)	1240.07	1123.1	5158.18	1393.4
SKSC_01_0193	100	1	***SLC3A1*** c.1366C>T, p.(Arg456Cys)	none	NA	2011.07	813.02	4191.65	1321.92
SKSC_04_0148	100	1	none	none	NA	9.08	32.52	56.82	22.59
SKSC_05_0022	100	1	***SLC3A1*** c.1400T>C, p.(p.Met467Thr)	***SLC3A1*** c.1011>A,p.(p.Pro337Pro)		1167.02	596.97	2607.32	1004.43
SKSC_06_0010	100	1	***SLC7A9*** c.544G>A, p.(Ala182Thr)	***SLC7A9*** c.505_508delAGCG, p.(p.Ser169fs)	yes	1167.02	596.97	2607.32	1004.43
SKSC_05_0024	80	1	***SLC3A1*** c.1400T>C, p.(p.Met467Thr)	***SLC3A1*** c.1424C>T, p.(p.Thr475Ile)	yes	660.47	596.8	2101.5	1032.07

The table indicates for each listed KSF (one per row) the % of cystine in the kidney stone, the variants identified in *SLC3A1* or *SLC7A9*, whether segregation *in trans* could be confirmed and the levels of amino acids in urine. Note that SKSC_05_0148 had no increased excretion of urinary amino acids and no variants in *SLC3A1* or *SLC7A9* so that we suspect the stone composition to be wrongly reported as being 100% cystine.

Supplemental Table 7: Stone recurrence analysis- Cox Proportional Hazards regression model hazard ratio and p-values

Phenotype	***Hazard ratio (95% CI)***	***P value***
Group (Monogenic KSF)	*2.4506 (1.1939 - 5.030)*	*0.015*
Age	*0.9883 (0.9675 - 1.010)*	*0.278*
Gender (male)	*1.9447 (0.9304 - 4.065)*	*0.077*

Publication bibliography

Lieske, John C.; Rule, Andrew D.; Krambeck, Amy E.; Williams, James C.; Bergstralh, Eric J.; Mehta, Ramila A.; Moyer, Thomas P. (2014): Stone Composition as a Function of Age and Sex. In *Clinical Journal of the American Society of Nephrology* 9 (12). Available online at https://journals.lww.com/cjasn/fulltext/2014/12000/stone_composition_as_a_function_of_age_and_sex.19.aspx.
